# Vitamin K2 Alleviates Insulin Resistance Associated Skeletal Muscle Atrophy via the AKT/mTOR Signalling Pathway

**DOI:** 10.1002/jcsm.13840

**Published:** 2025-06-04

**Authors:** Yingfeng Zhang, Yina Wang, Zhu Ming, Bin Li, Haitao Qi, Hongquan Xie, Guoliang Wang, Jiepeng Chen, Lili Duan, Ran Li, Ying Li

**Affiliations:** ^1^ Department of Nutrition and Food Hygiene School of Public Health, Harbin Medical University Harbin China; ^2^ Vitamin K2 Research Center Shenyang Pharmaceutical University Shenyang China; ^3^ Department of Clinical Nutrition Zigong First People's Hospital Zigong China

**Keywords:** AKT/mTOR pathway, insulin resistance, menaquinone‐7, skeletal muscle atrophy, vitamin K2

## Abstract

**Background:**

Skeletal muscle atrophy and insulin resistance (IR) aggravate each other. Vitamin K2 (VK2) exhibits beneficial effects on IR, but whether it improves IR associated skeletal muscle atrophy remains insufficiently understood. This study aims to investigate the effects of VK2 on IR associated skeletal muscle atrophy in high‐fat diet (HFD) mice and type 2 diabetes mellitus (T2DM) patients and explore the potential mechanisms.

**Methods:**

VK2 was administered to HFD‐fed C57BL/6 mice for 16 weeks. Grip strength, exercise capacity, oral glucose tolerance test (OGTT) and body fat rate were measured. Animals were sacrificed, and skeletal muscle and serum samples were collected to analyse muscle atrophy, glucose and lipid levels. The gene expression profile of skeletal muscle was determined by RNA sequencing. C2C12 cells were cultured for gene knockdown and overexpression experiments. For the randomized controlled trial (RCT), a total of 102 T2DM patients aged 50–80 years were recruited and randomly assigned to receive yogurt (one cup per day) with or without VK2 fortification (90 μg/day) for 6 months. Grip strength, skeletal muscle mass (SM), skeletal muscle mass index (SMI), 6‐m gait speed (6‐m GS), glycated haemoglobin (HbA1c), fasting blood glucose (FBG), fasting insulin (FINS) and homeostasis model assessment of insulin resistance (HOMA‐IR) were measured at 0, 3 and 6 months, respectively.

**Results:**

VK2 significantly improved grip strength (*p* < 0.01) and exercise capacity (all *p* < 0.05) in HFD‐fed mice. At the tissue level, VK2 increased skeletal muscle mass (*p* < 0.05) and cross‐sectional area of muscle fibres (*p* < 0.05), while reducing the proportion of fast‐twitch fibres (*p* < 0.01). VK2 treatment decreased body fat rate (*p* < 0.01) accompanied by enhanced whole‐body energy metabolism. VK2 also diminished the glucolipid metabolism parameters, including glucose (*p* < 0.01), HOMA‐IR (*p* < 0.01) and serum lipid levels. Regarding the mechanism, VK2 promoted the phosphorylation of proteins in the FAK‐AKT–mTOR‐P70S6K pathway by targeting Ccn2, thereby enhancing protein synthesis of C2C12 myotubes. In the RCT study, VK2 supplementation significantly increased grip strength (*p*_treatment × time_ = 0.017), SM (*p*_treatment × time_ = 0.001), SMI (*p*_treatment × time_ < 0.001) and decreased HbA1c (*p*_treatment × time_ < 0.001), FBG (*p*_treatment × time_ = 0.056), FINS (*p*_treatment × time_ < 0.001), and HOMA‐IR (*p*_treatment × time_ < 0.001) in T2DM subjects.

**Conclusions:**

Our findings demonstrated the beneficial effects of VK2 on insulin resistance related skeletal muscle atrophy by promoting protein synthesis via the AKT/mTOR pathway.

## 1 Introduction

Skeletal muscle atrophy, characterized by the loss of muscle mass and function, arises from numerous pathophysiological conditions, including aging, cancer, metabolic syndrome, diabetes and other metabolic diseases [1]. The associated prolonged illness recovery time, limitation of daily activities and decreased quality of life caused by muscle atrophy make it a critical global clinical issue [2].

Skeletal muscle represents over one‐third of body weight. It is also responsible for up to 80% of postprandial glucose uptake and utilization, playing a key role in insulin‐mediated glucose disposal [3]. When skeletal muscle atrophies, its inadequate response to insulin is associated with an increased risk of IR [4]. In turn, IR reduces glucose uptake and disrupts energy supply in skeletal muscle, leading to muscle atrophy [5]. Thus, the bidirectional relationship between skeletal muscle atrophy and IR highlights the importance of inhibiting muscle atrophy in managing and preventing metabolic diseases.

VK2, also known as menaquinones (MK‐n), is a fat‐soluble vitamin abundant in some fermented foods like natto and cheese and endogenously synthesized by gut microbiota [6]. Cohort studies indicated an inverse association between dietary VK2 intake levels and the risk of type 2 diabetes and metabolic syndrome [7, 8]. Correspondingly, it was reported that serum VK2 level was significantly lower in T2DM patients compared to non‐diabetic subjects [9]. Several RCT studies consistently demonstrated that VK2 supplementation alleviated IR and decreased glucose levels in T2DM patients [10, 11, 12]. Besides, animal and cellular studies have found that VK2 could alleviate IR by (1) enhancing the mitochondrial function of skeletal muscle through the SIRT1 [13], (2) regulating gut microbiota and its metabolites [14], (3) increasing the carboxylation of osteocalcin [15], and (4) inactivating the NF‐κB signalling pathway [16]. Therefore, adequate intake of VK2 is essential for populations with metabolic disorders, especially those with IR.

Although VK2 supplementation could improve IR, few studies have focused on its effects on IR‐related skeletal muscle atrophy. Cross‐sectional studies showed that increased dp‐ucMGP level, a VK2 deficiency marker in circulation, was linked to reduced axial skeletal muscle mass [17] and lower handgrip strength [18]. These results highlight the importance of VK2 nutritional status for skeletal muscle health. However, regarding the effects of VK2 supplementation on skeletal muscle, although one study has been conducted in patients with polycystic ovary syndrome [19], there are virtually no related studies in T2DM. Therefore, based on current evidence, it remains unclear whether VK2 can ameliorate IR‐related skeletal muscle atrophy, and the underlying mechanisms remain unexplored.

Here, to investigate the effects of VK2 on skeletal muscle atrophy and explore the underlying mechanisms, we first established chronic mouse models with HFD‐induced IR and skeletal muscle atrophy. Then, differentiated C2C12 myotubes served as the cell model in vitro. In addition, we performed a 6‐month RCT to validate the effects of VK2 supplementation on skeletal muscle atrophy in T2DM patients.

## 2 Methods

### 2.1 Mice

Male C57BL/6 mice (8 weeks old) were randomly divided into four groups: NC group—chow diets; HF group—60% HFD; LVK group—60% HFD combined with VK2 (MK‐7, Sigma‐Aldrich, #1381119) at 50 μg/kg body weight (bw); HVK group—60% HFD combined with VK2 at 2 mg/kg bw. The mice were fed a high‐fat diet for 16 weeks to establish chronic mouse models of IR‐related skeletal muscle atrophy. The VK2 was supplemented by gavage every other day, and the control groups were given corresponding amounts of vehicle.

The choice of dose was based on our previous RCT study [20]. Correction factors (K_m_) [21], frequency of gavage and the half‐life of VK2 were considered in dose calculation (50 μg/kg bw). The much higher dose (2 mg/kg bw) was administered to assess the safety of the VK2. The details of dose calculation were presented in the supplementary materials.

### 2.2 Grip Strength Test

An electronic dynamometer was used to measure grip strength (YIYAN YLS‐13A, China). Mice were placed on the dynamometer with forelimbs grabbing the metal strip and gently pulling back the tail in the horizontal direction, then increasing the force gradually until the mice were removed. Four measured values were recorded and averaged.

### 2.3 Treadmill Exhaustion Test

Before testing, mice were trained to run for 3 days (3–10 m/min, 10 min). During testing, the initial speed was set at 3 m/min and increased by 1.8 m/min every 3 min, up to 20 m/min. The speed was maintained until exhaustion. An electric grid was placed at the back of the treadmill, delivering small electric shocks of a constant intensity (0.2 mA) to motivate the mice to run.

### 2.4 Rotarod Test

Mice were placed on the rotarod at a constant speed (4 rpm) for 1 min as a habituation trial. When testing, record the time and speed when mice fall from an accelerating rotarod (4–30 rpm over 5 min). Results were the average of three trials, and there was a 30‐min rest period between each trial.

### 2.5 Comprehensive Laboratory Animal Monitoring System (CLAMS)

The CLAMS (Columbus Instruments, United States) was used to measure whole‐body energy metabolic parameters, including the respiratory exchange ratio (RER), energy expenditure and activity. RER and energy expenditure data were collected every 14 min. Activity data were measured every minute. Mice were measured over two consecutive days, and the final 24‐h period data (8 am to 8 am) were used for analysis.

### 2.6 Oral Glucose Tolerance Test (OGTT)

Mice were fasted for 12 h before the OGTT. Glucose solution (2 g/kg bw) was administered by gavage. Glucose concentrations of the tail vein were measured with an Accu‐Check glucometer (Bayer) before (0 min) and after (15, 30, 60, 90 and 120 min) glucose gavage.

### 2.7 Biochemical Indicators Analyses

Mice were euthanized, and the blood samples were harvested. Serum glucose, triglyceride (TG), total cholesterol (TCHO), low‐density lipoprotein (LDL), alanine aminotransferase (ALT) and aspartate aminotransferase (AST) were tested by ROCHE Modular P800 automatic biochemical analyser (ROCHE Diagnostics, Germany). Serum insulin was measured by mouse insulin ELISA kit (Elabscience, China). TG and glycogen of tissues were measured by triglyceride assay kit (Nanjing Jiancheng, China) and glycogen assay kit (Solarbio, China), respectively.

### 2.8 Histology and Immunostaining

For haematoxylin and eosin (HE) staining, tissues were fixed, embedded and cut into sections (5 μm), then dewaxed, rehydrated with gradient ethanol, and stained with haematoxylin and eosin (Beyotime, C0105). For immunostaining, muscle sections (10 μm) and C2C12 myotubes were blocked and permeabilized, then incubated with primary and secondary antibodies, respectively. The antibody information and experimental details were presented in the supplementary materials. Immunofluorescence imaging was taken using a Nikon‐U fluorescence microscope (Nikon, Eclipse Ti). Giemsa staining of C2C12 myotubes was performed using Giemsa solution according to the manufacturer's protocol.

### 2.9 RNA‐Seq and RT‐qPCR

Total RNA was isolated from skeletal muscle using TRIzol reagent (Invitrogen). The library construction and sequencing were performed at Novogene (Tianjin, China). More details were presented in the supplementary materials. For RT‐qPCR, primers were chemically synthesized by Sangon Biotech Co., Ltd. (Shanghai, China). cDNA was generated with the TaqMan™ MicroRNA Reverse Transcription Kit (ThermoFisher). Real‐time PCR was conducted using the SYBR Green PCR Master Mix (ThermoFisher), and the comparative CT (2 ‐ΔΔCt) method was used for relative quantitation. Primer sequences are presented in Table S1.

### 2.10 C2C12 Cell Culture and Differentiation

C2C12 myoblast cells were obtained from the Shanghai Institute of Biochemistry and Cell Biology (SIBCB, China). The basal medium was Dulbecco's modified Eagle's medium (DMEM, Gibco) with 10% foetal bovine serum (Gibco) and 1% antibiotic‐antimycotic solution (Beyotime, C0222) for the cells. For cell differentiation, C2C12 myoblasts with 80–90% confluence were cultured in the DMEM with 2% horse serum (Gibco) for 5–6 days until they fused into myotubes. Cells were maintained at 37 °C and in a 5% CO_2_ atmosphere.

Differentiated C2C12 myotubes were transfected with siCcn2 or overexpression Ccn2 using Lipofectamine 3000 (Invitrogen, United States) for 12 h and then treated with 0.2 mM palmitic acid (PA) (Sigma‐Aldrich, P0500) for 24 h in the presence or absence of 75 μM VK2. VK2 was dissolved with absolute ethanol before addition to the cell culture medium (the end concentration of EtOH was less than 0.4%) and the corresponding amounts of EtOH were added to the control.

### 2.11 Western Blotting Analysis

Total protein was extracted and quantitated. Protein samples were separated on 6–10% Bis‐Tris protein gels (Epizyme Biotech, China) and transferred onto polyvinylidene fluoride membranes (PVDF, Millipore, Germany). Membranes were blocked and then incubated with primary antibodies and secondary antibodies. All blots were imaged using the FluorChem system (Bio‐Techne). The antibody information and experimental details were presented in the supplementary materials.

### 2.12 Measurements of Protein Synthesis

The surface sensing of translation (SUnSET) method was used to measure the C2C12 myotube protein synthesis [22]. Briefly, cells were cultured in a medium containing 10 μg/mL puromycin (MCE) for 30 min at 37 °C after being treated with PA or VK2, and the protein samples were harvested for western blotting analysis.

### 2.13 Randomized Controlled Trial

#### 2.13.1 Participants

Participants with T2DM were recruited from the Nangang District Centers, Harbin, Heilongjiang Province. Inclusion criteria were as follows: aged from 50 to 80 years old, being diagnosed with T2DM and HbA1c ≥ 6.5%. Participants were excluded if they had taken vitamin K in the past 6 months; vitamin K antagonists within 1 year, such as warfarin; other supplements or nutrients for protecting against loss of muscle mass; with severe inconvenience in daily activities; and history of malignancy, musculoskeletal disease, or other diseases that may affect VK2 absorption and metabolism. A total of 121 subjects were recruited and 102 subjects met the inclusion criteria. Written informed consent was obtained from each participant before entering this study. The study was approved by the Ethical Committee of Harbin Medical University and registered at https://www.chictr.org.cn (ChiCTR1800019663).

#### 2.13.2 Study Design and Intervention

This study was a 6‐month randomized, controlled and double‐blind trial. Subjects were randomly assigned to either the VK2 group or the control group. Yogurt was selected as a matrix for VK2 fortification due to its recognized health benefits and because it also serves as an ideal matrix for nutrient fortification [23]. The VK2 group and the control group received yogurt (100 g/day, one cup) with and without fortified VK2 (MK‐7, 90 μg/d), respectively. The dose of VK2 was determined based on our previous RCT study [20]. Yogurt with similar appearance and taste was obtained from Sungen Bioscience Co., Ltd.

#### 2.13.3 Measurements

Body composition was measured by a bioelectrical impedance analyser (BIA, IoI353, JAWON, Korea). Skeletal muscle mass index (SMI) was skeletal muscle mass (kg) divided by the square of height (m). Grip strength was tested by a well‐calibrated hand dynamometer (EH101, Xiangshan, China), and the average value of three measurements was used for analysis. Physical performance was measured by 6‐m gait speed (6‐m GS). Participants were asked to go 6 m two times, and the average speed was calculated.

Blood samples were collected in the morning after 8 h of fasting at 0, 3 and 6 months for dp‐ucMGP, HbA1c, FBG and FINS measurements. Serum dp‐ucMGP was measured by ELISA kits (Jiangsu Enzyme‐Free Industrial Co. LTD). HbA1c was measured by an automatic glycohemoglobin analyser (HA‐8380, ARKRAY, Japan). FBG was detected by an automatic biochemical analyser (P800, Roche, Germany). FINS was measured by the Immunoassay System REV 4.1 (Beckman, USA). HOMA‐IR = fasting blood glucose (mmol/L) × fasting insulin (μU/mL)/22.5.

### 2.14 Statistical Analysis

For two groups, the Student's *t* test and chi‐square test were used for comparison. If the data were not normally distributed, the Wilcoxon rank sum test was applied. For multiple groups, the within‐group differences were analysed by one‐way ANOVA or Kruskal–Wallis. The effects of VK2 supplementation in T2DM patients were assessed by using linear mixed models with treatment, time and treatment × time interaction as fixed effects and participant included as a random effect. The *p* < 0.05 was considered statistically significant.

## 3 Results

### 3.1 VK2 Supplements Improve the Loss of Skeletal Muscle Function and Mass Induced by a High‐Fat Diet

**FIGURE 1:**
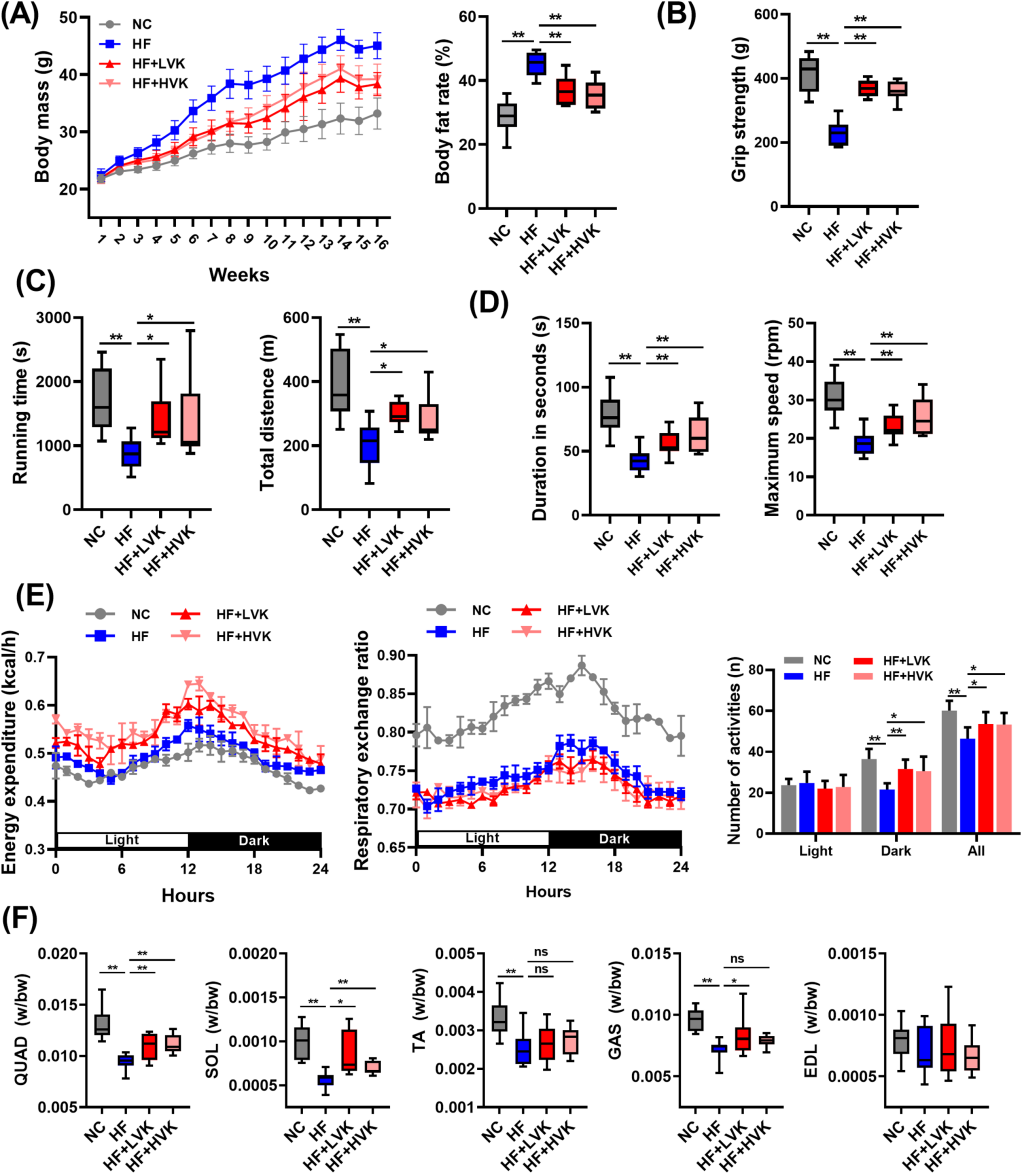
VK2 improved the loss of muscle function and mass induced by a high‐fat diet. (A) Body mass (g) and body fat rate (%) of mice, *n* = 10. (B) Forelimb grip strength (g), *n* = 10. (C) Running time (s) and total distance (m) in treadmill exhaustion test, *n* = 9. (D) Duration time (s) and maximum speed (rpm) in the rotarod test, *n* = 10. (E) Whole‐body metabolic analysis of energy expenditure (kcal/h), respiratory exchange ratio, and number of activities (*n*), *n* = 3. (F) Percentage of skeletal muscle weight in body weight (w/bw), *n* = 10. Quadriceps (QUAD), soleus (SOL), tibialis anterior (TA), gastrocnemius (GAS) and extensor digitorum longus (EDL). Ns, no significance, *p < 0.05, **p < 0.01.

After 16 weeks of intervention, the mice with VK2 treatment showed less weight gain and lower body fat rates compared with the HF group (Figure 1A). Compared to the HF group, grip strength and exercise capacity significantly increased in the LVK and HVK groups (Figure 1B–D). To determine the effects of VK2 on the whole‐body energy metabolism, energy expenditure, RER and activity of mice were monitored. The energy expenditure of the LVK and HVK mice was higher than that of HF mice (Figure 1E, Figure S1A). The decreased RER indicated a switch of the metabolic pattern from glucose metabolism to lipid metabolism in LVK and HVK mice (Figure 1E, Figure S1B). We also observed increased activity during nighttime hours in both LVK and HVK mice (Figure 1E). Consistent with muscle function, VK2 increased the relative mass (w/bw) of skeletal muscles, especially in QUAD and SOL muscles (Figure 1F).

### 3.2 VK2 Inhibits Muscle Fibre Atrophy and Alters Muscle Fibre Types in HFD‐Fed Mice

**FIGURE 2:**
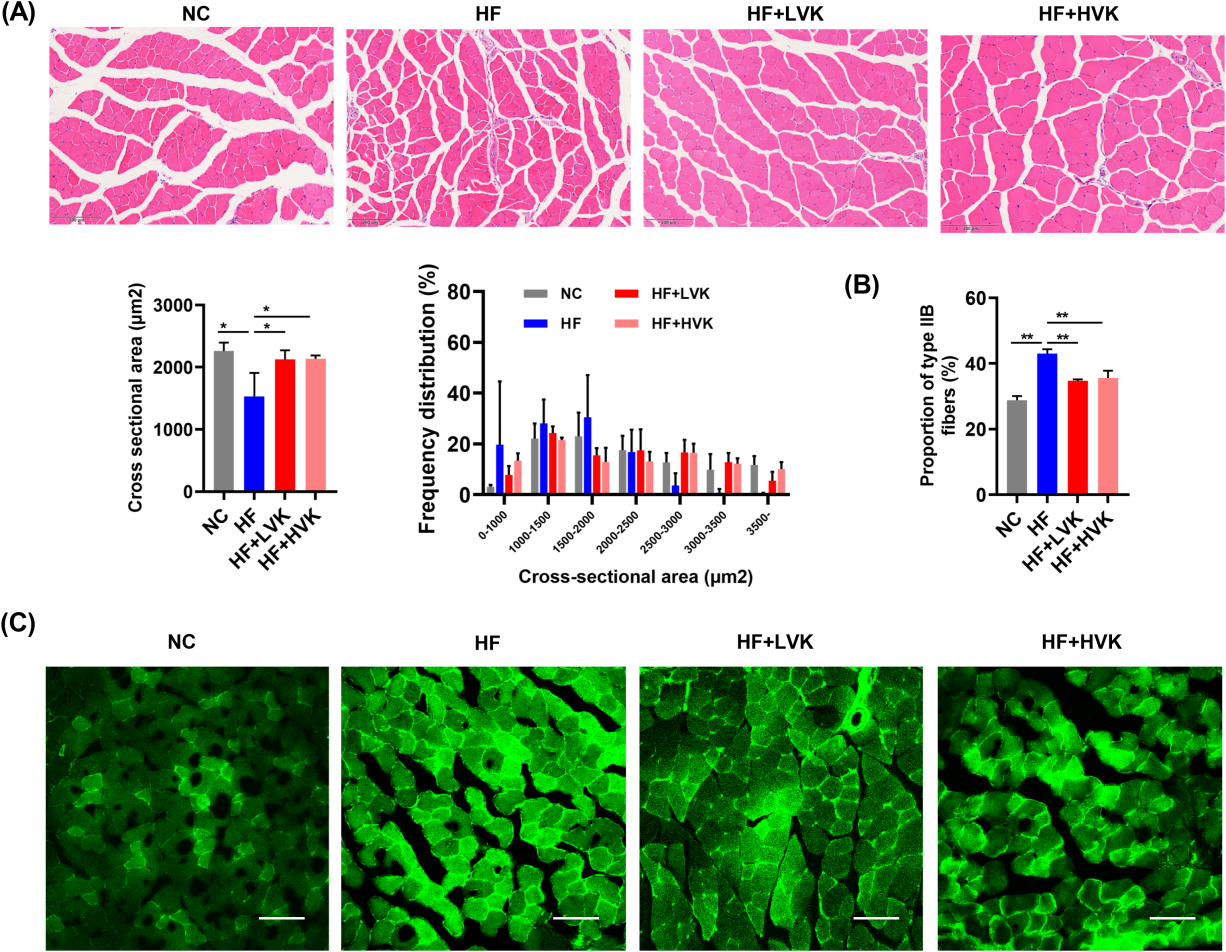
VK2 reduced skeletal muscle fibre atrophy and altered muscle fibre types in HFD‐fed mice. (A) Haematoxylin and eosin (HE) staining of skeletal muscle, *n* = 3. (B) Proportion of type IIB fibres in skeletal muscle (%). (C) Immunofluorescence staining of type IIB fibres in skeletal muscle, *n* = 3. *p < 0.05, **p < 0.01.

The muscle fibre cross‐sectional area was smaller after being fed with a high‐fat diet compared to NC mice, while VK2 supplements significantly reversed the atrophy of skeletal muscle (Figure 2A). VK2 decreased the proportion of type IIB fibres (Figure 2B,C), a subtype of fast‐twitch fibres characterized by lower mitochondrial content. We therefore examined the gene expression of mitochondrial transcription factor A (TFAM) and nuclear respiratory factor 1 (Nrf1), two transcriptional factors used as markers of mitochondrial biogenesis [24]. VK2 increased both TFAM and Nrf1 expression (Figure S2A, B). The lipid droplets in the liver and epididymal white adipose tissue (eWAT) in LVK and HVK mice were smaller than those in HF mice (Figure S2C). In addition, the weight of fat tissues all reduced in LVK and HVK mice (Figure S2D). The results indicated that VK2 improved skeletal muscle concomitantly with decreased lipid accumulation in HFD‐fed mice.

### 3.3 VK2 Alleviates IR and Glucolipid Metabolism Disorders in HFD‐Fed Mice

**FIGURE 3:**
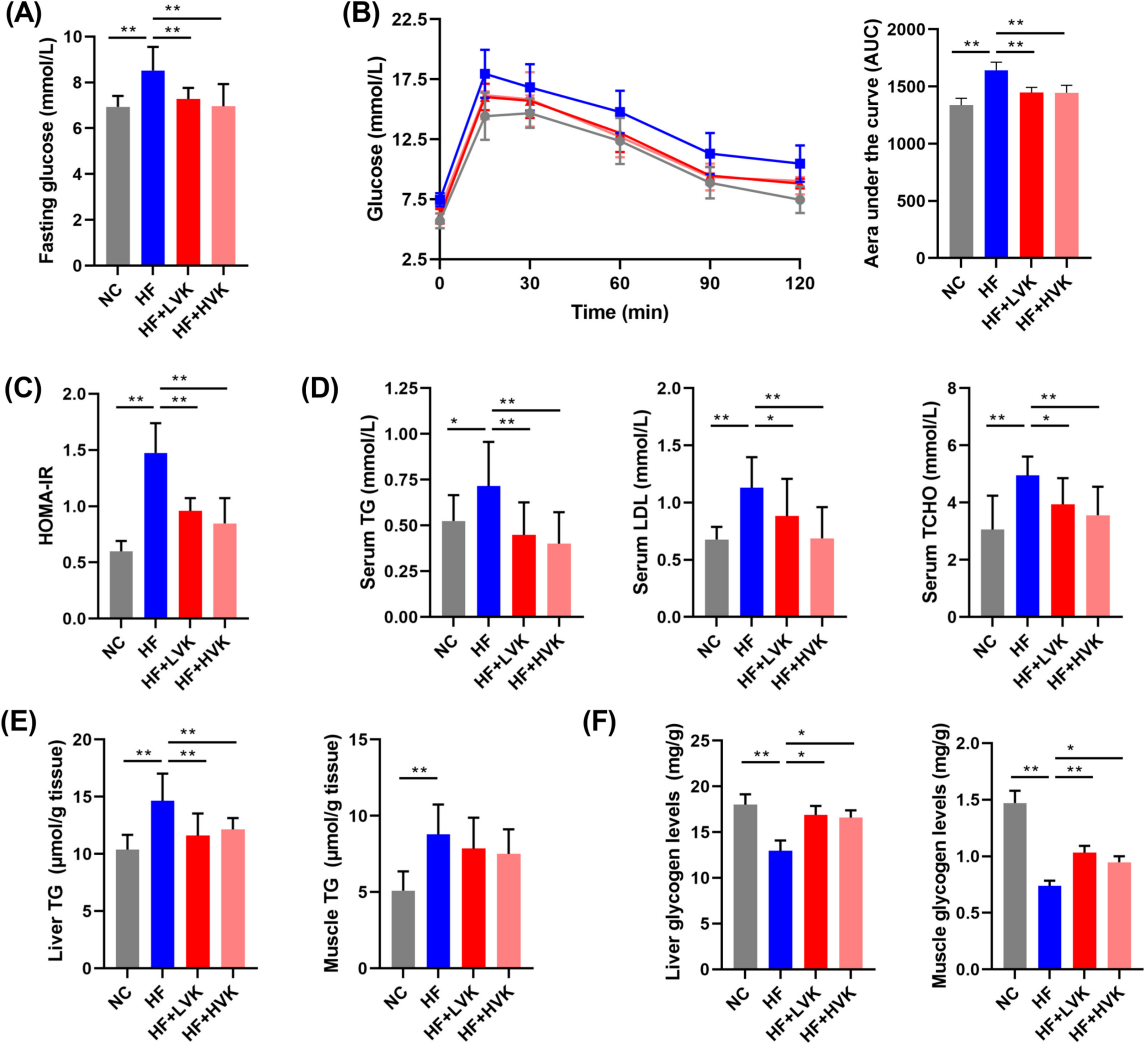
VK2 alleviated disorders in glucose and lipid metabolism. (A) Fasting glucose of mice (mmol/L), *n* = 10. (B) Glucose levels (mmol/L) and area under the curve (AUC) in oral glucose tolerance test (OGTT), *n* = 8. (C) Homeostasis model assessment of insulin resistance (HOMA‐IR), *n* = 10. (D) Serum triglyceride (TG), low density lipoprotein (LDL) and total cholesterol (TCHO) levels (mmol/L), *n* = 10. (E) Liver and skeletal muscle TG levels (μmol/g), *n* = 7. (F) Liver and skeletal muscle glycogen levels (mg/g), *n* = 7 and 8, respectively. *p < 0.05, **p < 0.01.

At 16 weeks, the fasting glucose levels were significantly lower in LVK and HVK mice (Figure 3A). In the OGTT trial, oral supplementation with VK2 had a significant improvement in glucose tolerance (Figure 3B). Serum insulin and HOMA‐IR were also significantly reduced in the LVK and HVK mice compared to HF mice (Figure 3C, Figure S3B). In addition, oral administration of VK2 prevented serum TG, LDL and TCHO levels from rising in HFD‐fed mice (Figure 3D), concurrent with decreased fat accumulation and increased storage of glycogen in the liver and skeletal muscle (Figure 3E,F). Serum ALT and AST also decreased in the LVK and HVK mice (Figure S3C, D).

### 3.4 The Gene Expression Profiles Are Changed by the VK2 Supplementation in HFD‐Fed Mice

**FIGURE 4:**
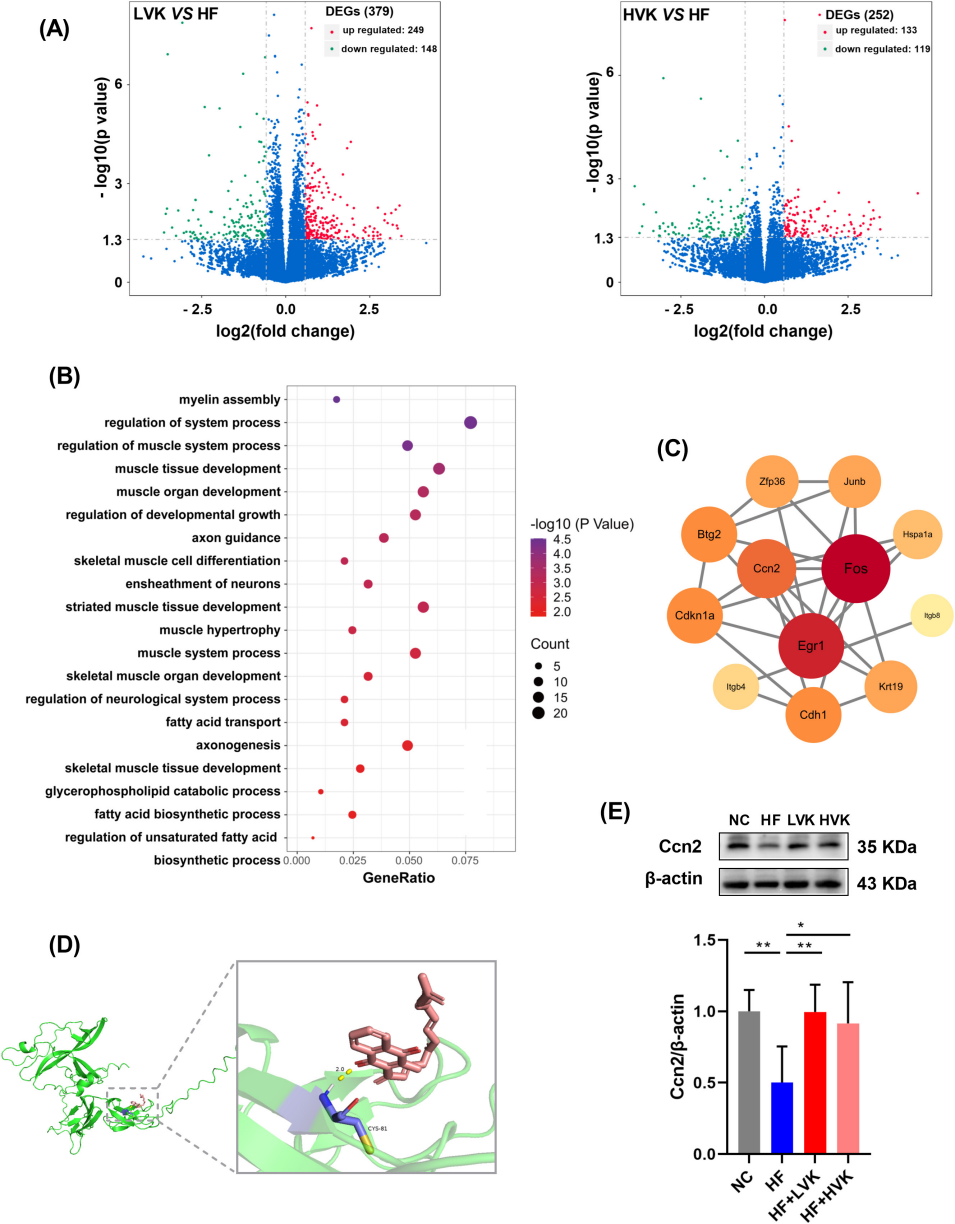
The gene expression profile of skeletal muscle was changed by VK2 supplementation in HFD‐fed mice. (A) The differentially expressed genes (DEGs) between the LVK, HVK and HF groups, *n* = 5. (B) GO analysis of DEGs in the LVK group in skeletal muscle. (C) The PPI analysis of rescue genes set in the LVK group. (D) Binding model between vitamin K2 and Ccn2 by molecular docking, the binding free energy is −4.04 kcal/mol. (E) Western blotting and quantitative analysis of the levels of Ccn2 in skeletal muscle, *n* = 5. *p < 0.05, **p < 0.01.

RNA sequencing on the skeletal muscle tissue of mice was performed to further study the molecular mechanism behind these changes. VK2 supplements resulted in a large number of differentially expressed genes (DEGs) between the VK and HF groups (Figure 4A, Figure S4A). The results of Gene Ontology (GO) analysis showed that the related pathways enriched in DEGs were involved in muscle tissue development and muscle hypertrophy (Figure 4B). Intriguingly, more pathways related to skeletal muscle development were enriched in the LVK group than in the HVK group (Figure S4B). Therefore, we implemented further exploration in the LVK group. To check out the potential core targets regulated by VK2, we created gene sets of DEGs between the NC and HF mice that were significantly rescued in LVK mice (Figure S5A). The hub genes of PPI analysis included FOS, Egr1, Ccn2, Cdkn1a, etc. (Figure 4C, Figure S5B). RT‐PCR and molecular docking were used for further screening of the potential core targets, and the results of cellular communication network factor 2 (Ccn2) suggested a high possibility of being regulated by VK2 (Figure 4D, Figure S5C). The protein level of Ccn2 was also markedly up‐regulated in LVK and HVK mice (Figure 4E).

### 3.5 VK2 Regulates the Phosphorylation of FAK and Its Downstream Pathways Through Targeting Ccn2

**FIGURE 5:**
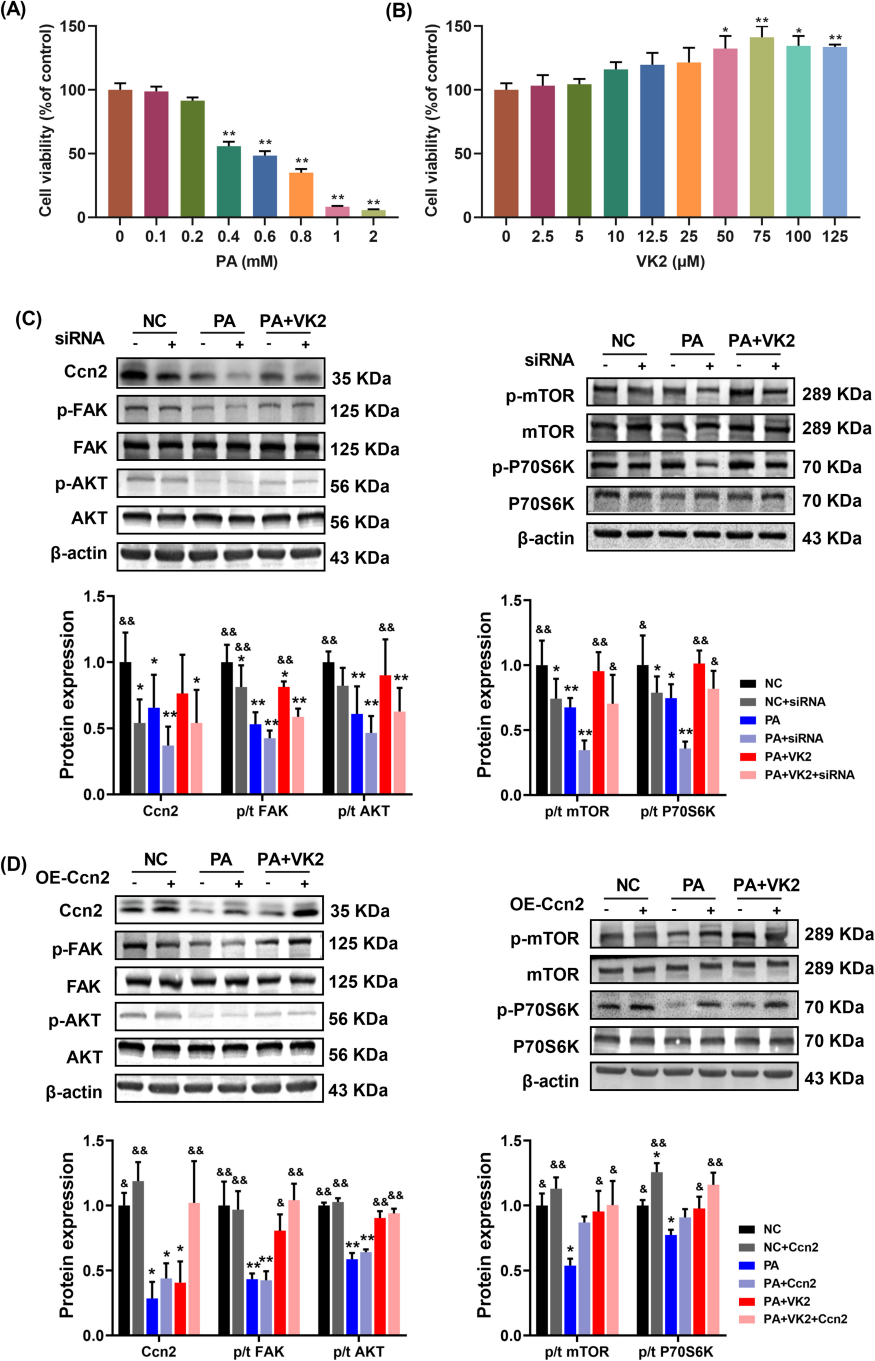
VK2 regulated the phosphorylation of FAK and its downstream pathways through Ccn2. (A), (B) Results of cell viability measured by Cell Counting Kit‐8 (CCK8) tests under the different concentrations of PA (A, *n* = 6) and VK2 (B, *n* = 5) (% of control). (C,D) Western blotting and quantitative analysis of the levels of Ccn2, FAK, p^Tyr397^FAK, AKT, p^S473^AKT, mTOR, p^s2448^mTOR, P70S6K, p^Thr389^P70S6K and β‐actin in C2C12 myotubes with knockdown (C) or overexpression (D) of Ccn2 (normalised to NC), *n* = 4. For A and B, *p < 0.05, **p < 0.01 vs. the control group (0 mM or μM). For others, *p < 0.05 vs. NC, **p < 0.01 vs. NC; ^&^ *p* < 0.05 vs. HF, ^&&^ *p* < 0.01 vs. HF.

According to the results of the CCK8 assay, C2C12 myotubes were exposed to 0.2 mM of PA in the presence or absence of 75 μM VK2 (Figure 5A,B). Previous studies found that Ccn2 knockout impaired the activity of focal adhesion kinase (FAK) [25], and FAK could regulate protein synthesis via FAK‐AKT signalling axes [26, 27]. Therefore, we hypothesized that VK2 might potentially interact with Ccn2 to modulate the FAK‐AKT signalling pathway. As illustrated in Figure 5C, Ccn2 knockdown led to a reduction in the phosphorylation of proteins involved in FAK‐AKT–mTOR‐P70S6K signalling in differentiated C2C12 myotubes. Compared to the PA+VK2 group, the phosphorylation levels of proteins were significantly lower in the PA+VK2+siRNA group (Figure 5C). Conversely, Ccn2 overexpression increased the phosphorylation of FAK and downstream pathway proteins. However, the phosphorylation levels of proteins in the PA+Ccn2 group were still lower than the PA+VK2 group (Figure 5D). These findings indicated that VK2 could regulate the phosphorylation of the FAK‐AKT–mTOR‐P70S6K pathway through interaction with Ccn2.

### 3.6 VK2 Promotes Protein Synthesis in C2C12 Myotubes

**FIGURE 6:**
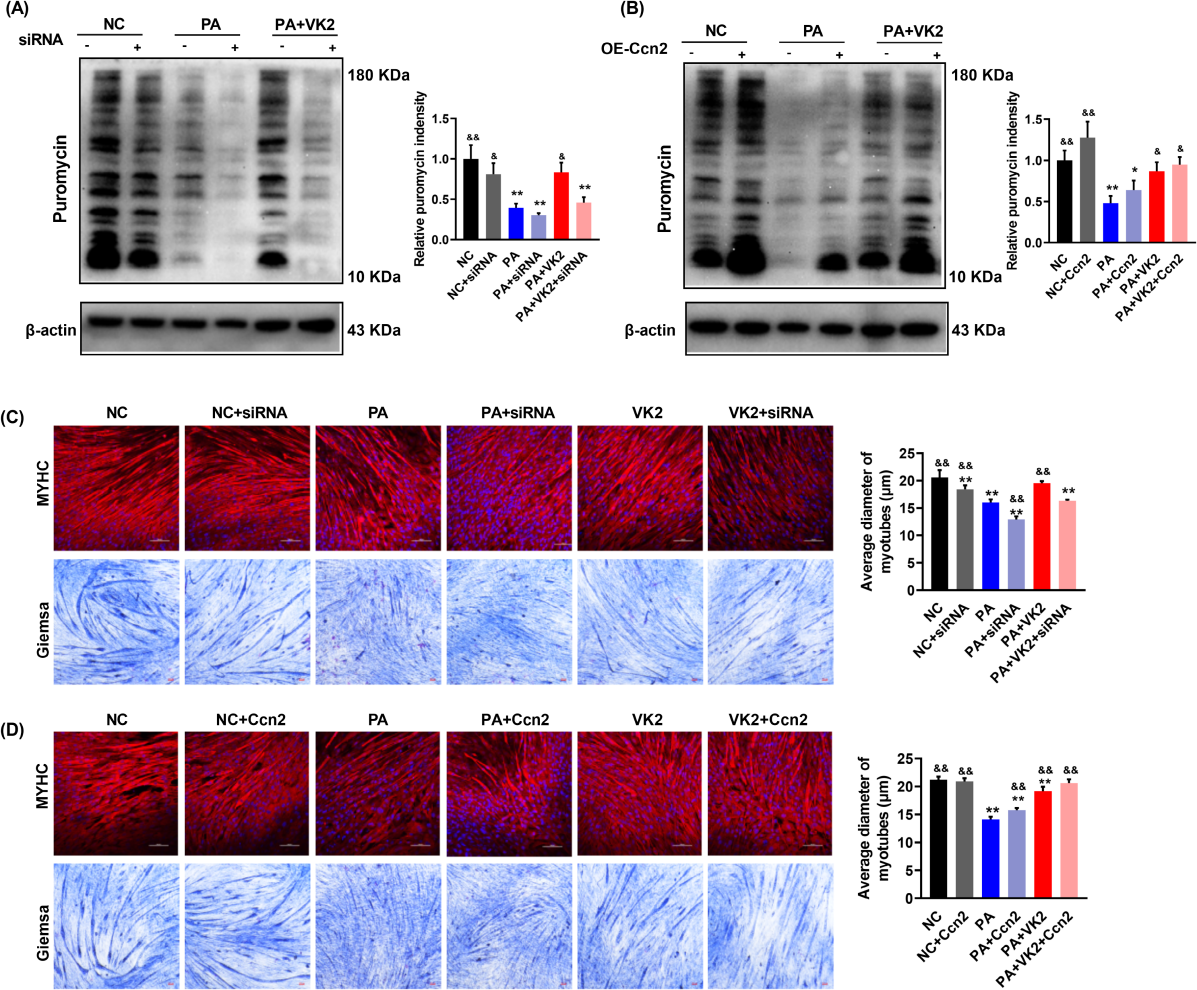
VK2 promotes protein synthesis in C2C12 myotubes through Ccn2. (AB) Western blotting and quantitative analysis of puromycin incorporation in surface sensing of translation (SUnSET) assay with knockdown (A) or overexpression (B) of Ccn2, *n* = 4. (C,D) Immunofluorescence staining and Giemsa staining of myotubes with knockdown (C) or overexpression (D) of Ccn2, *n* = 4. Red and blue indicated MYHC and DAPI staining, respectively. *p < 0.05 vs. NC, **p < 0.01 vs. NC; ^&^ *p* < 0.05 vs. HF, ^&&^ *p* < 0.01 vs. HF.

We next investigated the protein synthesis efficiency in C2C12 myotubes using the SUnSET method. In line with the reduced phosphorylation of ribosomal protein S6 kinase (P70S6K), the Ccn2 knockdown decreased the protein synthesis efficiency. VK2 did not enhance protein synthesis efficiency with the Ccn2 knockdown in the PA+VK2+siRNA group (Figure 6A). While overexpressing Ccn2, the protein synthesis efficiency in the PA+Ccn2 group remained lower than that in the PA+VK2 group (Figure 6B). To assess cellular growth, immunostaining and Giemsa staining were employed to examine the morphological characteristics of C2C12 myotubes. The Ccn2 knockdown abolished the VK2‐mediated increase in myotube diameter **in the** PA+VK2+siRNA group (Figure 6C). Similarly, Ccn2 overexpression did not reverse the PA‐induced decrease in myotube diameter in the PA+Ccn2 group, although there was a slight increase compared to the PA group (Figure 6D). Collectively, these results suggested that VK2 treatment might mitigate the PA‐induced reduction in protein synthesis and subsequent cellular growth by targeting Ccn2.

### 3.7 VK2 Supplementation Improved Grip Strength and Skeletal Muscle Mass, as Well as Decreasing Glucose Level and IR in T2DM Patients

A randomized controlled study was conducted to verify the effects of VK2 in T2DM patients. Throughout the 6‐month intervention, a total of 93 participants completed the trial (Figure S6). The baseline characteristics were not significantly different between the two groups (Table 1). The rate of yogurt intake was 96.7%, indicating good participant compliance. The dp‐ucMGP levels of the VK2 and placebo groups were 5.78 ± 0.65 and 6.09 ± 0.49 pmol/L, respectively. The VK2 supplementation brought a corresponding significant reduction in dp‐ucMGP level compared to placebo (−0.33 ± 0.41 pmol/L vs − 0.11 ± 0.18 pmol/L, *p* = 0.001). No adverse effects were observed in this study.

**TABLE 1:** Baseline characteristics of participants.

Variable	VK2 group (*n* = 56)	Control group (*n* = 46)	*p*
**Age (years)**	62.68 ± 7.13	63.36 ± 6.41	0.612
**Male, *n* (%)**	30 (53.57%)	22 (47.82%)	0.564
**Height (cm)**	163.80 ± 8.42	164.37 ± 9.26	0.750
**Weight (kg)**	67.32 ± 10.56	68.86 ± 13.69	0.529
**BMI (kg/m** ^**2**^ **)**	25.08 ± 2.85	25.37 ± 3.73	0.653
**Body fat rate (%)**	28.41 0.05	29.10 ± 0.06	0.535
**WHR**	0.90 ± 0.11	0.92 ± 0.06	0.229
**Duration of diabetes ≥ 10 years, *n* (%)**	31 (55.35%)	24 (52.17%)	0.748
**Energy intake (kcal/day)**	1356.87 ± 697.66	1189.19 ± 705.33	0.360
**Protein intake (g/day)**	58.34 ± 32.75	51.21 ± 35.58	0.121
**Fat intake (g/day)**	47.67 ± 29.95	40.98 ± 26.99	0.572
**Smoking, *n* (%)**	6 (10.71%)	9 (19.57%)	0.188
**Drinking, ** ***n* (%)**	18 (32.14%)	10 (21.73%)	0.272
**Regular exercise, *n* (%)** ^a^	30 (53.57%)	31 (67.39%)	0.157
**SBP (mmHg)**	134.36 ± 15.58	134.61 ± 16.93	0.380
**DBP (mmHg)**	78.19 ± 10.36	78.87 ± 9.38	0.220
**dp‐ucMGP (pmol/L)** ^b^	6.12 ± 0.65	6.20 ± 0.47	0.890

*Note:* Values are means ± SDs for continuous variables or *n* (%) of subjects for categorical variables.

Abbreviations: BMI, body mass index; DBP, diastolic blood pressure; SBP, systolic blood pressure; WHR, the ratio of waist to hip.

a Regular exercise, more than 3 times a week.

b dp‐ucMGP concentrations were natural log transformed.

**FIGURE 7:**
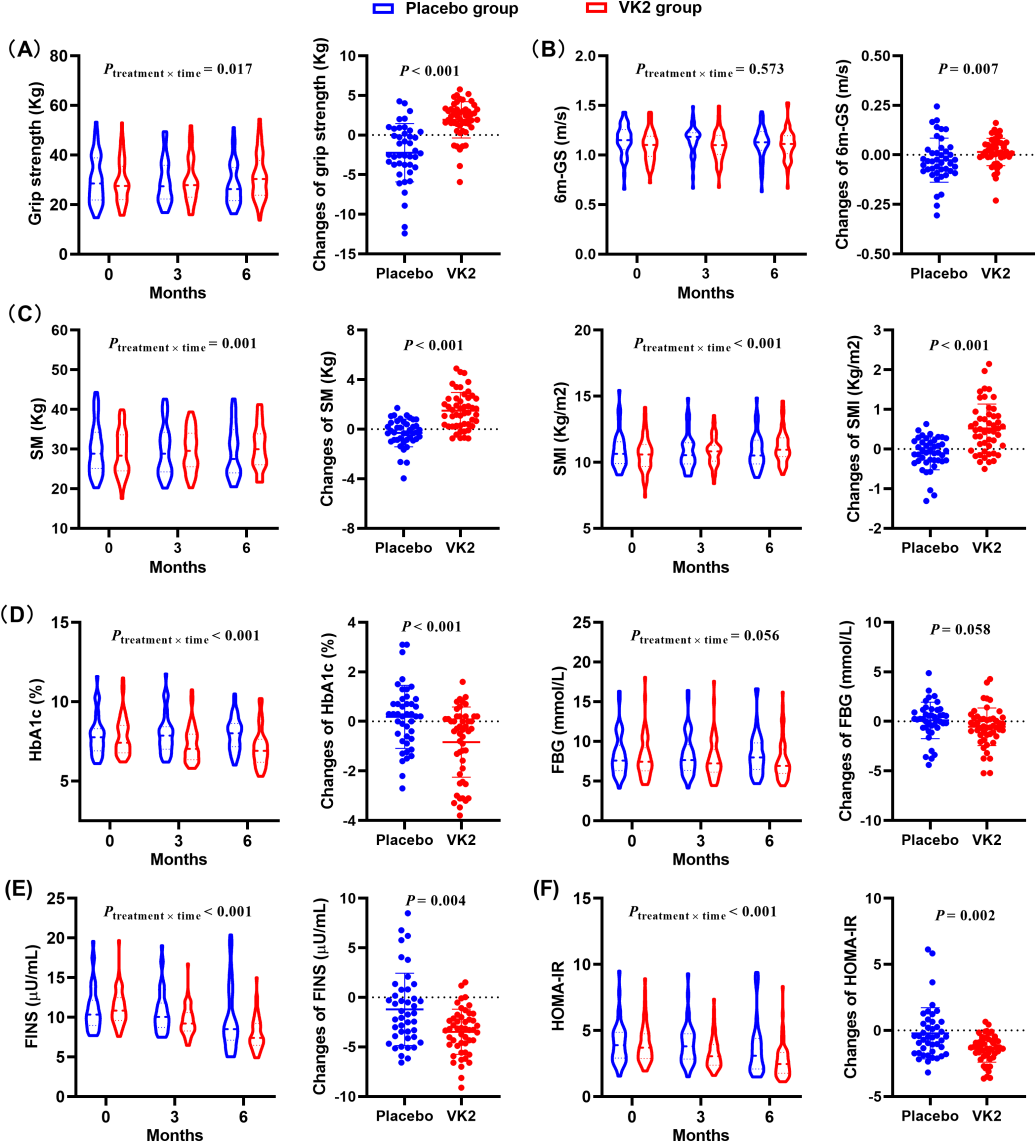
The effects of VK2 supplementation on skeletal muscle and biochemical indicators in T2DM subjects. (A) Grip strength (kg). (B) Six‐meter gait speed (6 m‐GS) (m/s). (C) Skeletal muscle mass (SM) (kg) and skeletal muscle mass index (SMI) (kg/m^2^). (D) Glycated haemoglobin (HbA1c) (%) and fasting blood glucose (FBG) (mmol/L). (E) Fasting insulin (FINS) (μU/mL). (F) Homeostasis model of assessment insulin resistance (HOMA‐IR). *P*_treatment × time_ values were based on a linear mixed model (placebo group, *n* = 43; VK2 group, *n* = 50). The changes refer to the differentials from 0 to 6 months. *p* Values were assessed by ANOVA (changes of SM, HbA1c and FBG) and Wilcoxon test (changes of grip strength, 6 m‐GS, SMI, FINS and HOMA‐IR).

After the 6‐month intervention, compared with the placebo group, grip strength increased significantly in the VK2 group (*p*_treatment × time_ = 0.017) (Figure 7A). No significant difference was observed in 6 m‐GS (*p*_treatment × time_ = 0.573) between the two groups, but the gait speed decreased less after VK2 supplementation (*p* = 0.007) (Figure 7B). What is more, the SM and SMI were both increased in the VK2 group while decreasing in the placebo group (*p*_treatment × time_ = 0.001 and *p*_treatment × time_ < 0.001, respectively) (Figure 7C). Consistently, in the VK2 group, the HbA1c decreased but increased in the placebo group (*p*_treatment × time_ < 0.001) (Figure 7D). The FBG showed a decreasing trend in the VK2 group, but the overall comparison (*p*_treatment × time_ = 0.056) and the differences between the two groups (*p*_treatment × time_ = 0.058) only showed borderline statistical significance (Figure 7D). The FINS and HOMA‐IR were also decreased significantly in the VK2 group (both *p*_treatment × time_ < 0.001) compared with the placebo group (Figure 7E,F).

## 4 Discussion

IR is closely associated with a decline in skeletal muscle mass and function [28]. Treatment with VK2 significantly alleviated skeletal muscle atrophy as well as improving IR and glucolipid disorders in HFD‐fed mice. Mechanistically, we found that VK2 enhanced protein synthesis in skeletal muscle cells by modulating the FAK‐AKT–mTOR‐P70S6K pathway by targeting Ccn2. Furthermore, an RCT conducted in T2DM patients also validated that VK2 supplementation increased skeletal muscle mass and strength while reducing HOMA‐IR and blood glucose levels. These findings collectively underscored the potential of VK2 in mitigating IR‐related skeletal muscle atrophy by promoting protein synthesis.

Insulin resistance and skeletal muscle atrophy are often considered “two sides of the same coin”, especially in patients with metabolic disorders, as they often interact to exacerbate disease progression [29]. Several previous studies have indicated the beneficial effects of VK2 on IR, including increased insulin sensitivity [12, 14] and reduced FBG and HbA1c in T2DM subjects [10, 11]. Consistently, our results showed that VK2 effectively reduced fasting glucose, increased glucose tolerance, and decreased the HOMA‐IR in HFD‐fed mice and T2DM patients. VK2 also promoted the uptake and utilization of peripheral glucose and lipid by the liver and skeletal muscle in HFD‐fed mice.

Improvements in IR generally indicate a positive shift in the overall metabolic state. In this study, VK2 significantly increased the whole‐body energy metabolism of the HFD‐fed mice. Mitochondria play a central role in cellular energy metabolism, and VK2 has been reported to maintain normal ATP production by acting as a mitochondrial electron carrier [30]. Moreover, VK2 could activate SIRT1 to increase mitochondrial biogenesis and improve mitochondrial dysfunction [13]. Our study also observed that VK2 increased the two markers of mitochondrial biogenesis, TFAM and Nrf1. Together, these findings suggested that VK2 ameliorated the overall metabolic disorders associated with IR. Currently, improving skeletal muscle atrophy in metabolic disorders by targeting IR is a candidate [31], but whether VK2 contributes to ameliorating IR‐related skeletal muscle atrophy remains unclear.

In this regard, we administered VK2 to HFD‐fed mice and observed improvements in exercise capacity. In T2DM patients, we observed an increase in grip strength after 6 months of VK2 supplementation (90 μg/day). Skeletal muscle function is directly affected by its mass, which was also significantly increased in HFD‐fed mice and T2DM patients. Consistently, a study in 84 young women with polycystic ovary syndrome showed that VK2 supplementation (90 μg/d) significantly improved skeletal muscle mass [19]. Another trial conducted in 60 middle‐aged T2DM patients found no changes in total body muscle mass after VK2 supplementation (200 μg/day) [32]. Despite the use of a higher dose, no significant changes were observed in the levels of dp‐ucMGP, a biomarker widely regarded as indicative of VK2 supplementation efficacy, suggesting poor compliance among the subjects. In addition, a study conducted in 80 elderly men (77 ± 5 years) found no improvement in grip strength or physical performance after 6 months of VK2 supplementation (100 μg/day) [33]. The results from non‐diabetic subjects further suggest that the effects of VK2 on skeletal muscle and IR are complementary. Given the inconsistent findings, the benefits of VK2 on IR‐related skeletal muscle atrophy should be emphasized.

Skeletal muscle atrophy is often accompanied by histopathological alterations, mainly changes in the size and type of muscle fibres, with little change in the number. The cross‐sectional area of muscle fibres was increased in HFD‐fed mice supplemented with VK2, aligning with the increase in skeletal muscle mass. Compared with the slow‐twitch fibres, fast‐twitch muscle fibres have fewer aerobic enzymes and mitochondria, resulting in weaker fatigue resistance [34]. VK2 reduced the proportion of fast‐twitch muscle fibres by improving mitochondrial function [35]. Consistently, we found that VK2 decreased the ratio of type IIB fibres, a main type of fast‐twitch fibre in skeletal muscle. Therefore, based on the improvements in skeletal muscle fibre size and type, VK2 attenuated IR‐related skeletal muscle atrophy.

To further explore the molecular changes induced by VK2 supplementation, we performed RNA sequencing in skeletal muscle. Our results showed that VK2 altered the gene expression profiles in HFD‐fed mice. Through a series of analyses, we narrowed our focus to the gene Ccn2. Ccn2 promoted skeletal muscle hypertrophy by upregulating FAK [25]. Increasing FAK promoted protein synthesis via the AKT/mTOR signalling pathway [26, 27], which is crucial for maintaining the balance between protein synthesis and degradation in skeletal muscle [36]. Moreover, as another kind of vitamin K, vitamin K1 was found to upregulate this pathway, thereby partially protecting skeletal muscle from lipopolysaccharide‐triggered damage [37]. However, vitamin K1 is primarily distributed in the liver, whereas vitamin K2 is the predominant form transported into skeletal muscle [38]. Therefore, we speculated that VK2 might regulate the AKT/mTOR pathway to improve skeletal muscle atrophy by targeting Ccn2 in skeletal muscle.

The AKT/mTOR signalling pathway is involved in some treatments for skeletal muscle atrophy [1, 2]. For example, magnesium supplementation accompanied by low‐magnitude high‐frequency vibration significantly attenuated the sarcopenic muscle atrophy through the AKT/mTOR pathway in accelerated aging mice [39]. Codonopsis lanceolata, a medicinal plant, also activated the AKT/mTOR pathway to enhance muscle protein synthesis and ameliorate HFD‐induced skeletal muscle atrophy [40]. Despite differences in animal models and treatments, the involvement of AKT/mTOR upregulation is consistent. As expected, VK2 upregulated the phosphorylation of the FAK‐AKT–mTOR‐P70S6K pathway by targeting Ccn2 in differentiated C2C12 myotubes. This activation promoted protein synthesis and increased the diameter of myotubes. These findings indicated that VK2 could enhance protein synthesis, contributing to an increase in the size of skeletal muscle fibres and ultimately alleviating the loss of muscle mass and function associated with IR.

We confirmed previously documented benefits of VK2 on IR and further demonstrated its capacity to improve IR‐related skeletal muscle atrophy in HFD‐fed mice and T2DM patients. Nevertheless, considering the complexity of the occurrence and development of skeletal muscular atrophy, the muscular benefits of VK2 should be verified using other animal models, such as older age and genetically engineered models. In addition, VK2 exerts multiple benefits in mitigating an array of diseases, so it is essential to figure out how VK2 modulates Ccn2, whether it regulates transcription, directly binds to proteins of Ccn2, or other ways. Finally, given the relatively short duration and small sample size in the clinical trial, further research with larger populations and longer follow‐up periods is needed. Due to the significant heterogeneity in current RCT studies, additional research involving diverse populations and regions is necessary to reach a consensus on the effects of vitamin K2 on skeletal muscle atrophy.

## 5 Conclusion

In conclusion, VK2 improved IR and mitigated IR‐related skeletal muscle atrophy in HFD‐fed mice and T2DM subjects. Mechanistically, VK2 enhanced protein synthesis by modulating the FAK‐AKT–mTOR‐P70S6K pathway via targeting Ccn2. These findings suggest that VK2 supplementation could be a promising health‐protective strategy for individuals with insulin resistance.

## Ethics Statement

The study has been approved by the Ethical Committee of Harbin Medical University (HMUIRB2018RCT002) and was performed in accordance with the ethical standards as laid down in the 1964 Declaration of Helsinki and its later amendments or comparable ethical standards. All participants gave written informed consent before entering the study. All mouse manipulations were performed in accordance with procedures approved by the Animal Care Committee of Harbin Medical University.

## Conflicts of Interest

The authors declare no conflicts of interest.

## Supporting information


**Figure S1.** Vitamin K2 improved the whole‐body energy metabolism in high‐fat diet mice. (A) energy expenditure (kcal/h). (B) Respiratory exchange ratio, *n* = 3. *p < 0.05, **p < 0.01.
**Figure S2.** Vitamin K2 reduced lipid accumulation induced by high‐fat diet. (A) The relative mRNA expression of nuclear respiratory factor 1 (Nrf1), *n* = 4. (B) The relative mRNA expression of mitochondrial transcription factor A (Tfam), *n* = 4. (C) HE staining of liver and epididymal white adipose tissue (eWAT). (D) The weight of epididymal fat, inguinal fat, perirenal fat, and brown fat tissues (g), *n* = 10. *p < 0.05, **p < 0.01.
**Figure S3.** VK2 alleviated disorders in glucose and lipid metabolism. (A) Fasting glucose at 8 weeks of intervention (mmol/L), *n* = 10. (B) Serum insulin levels (ng/mL), *n* = 10. (C) Serum alanine aminotransferase (ALT) levels (U/L), *n* = 10. (D) Serum aspartate aminotransferase (AST) levels (U/L), *n* = 10. *p < 0.05, **p < 0.01.
**Figure S4.** (A) The number of differentially expressed genes in the overlap between gene sets of DEGs in the LVK and HVK group in skeletal muscle. (B) GO analysis of DEGs in the HVK groups in skeletal muscle.
Figure S5 The analysis and screening of target genes modulated by vitamin K2. (A) Definitions of rescue genes. (B) Target genes identified through PPI and Cytoscape analysis. Four different topological analysis methods, degree, MCC, MNC and EPC were used to extract hub genes by cytoHubba module in Cytoscape. (D) The relative mRNA expression of potential hub genes, *n* = 4. *p < 0.05, **p < 0.01.
**Figure S6.** Study profile of the randomized controlled trial in T2DM subjects.
**Figure S7.** Vitamin K2 improved skeletal muscle mass and exercise capacity in high‐fat diet induced IR mice while reducing IR and glucolipid disorders. The probable mechanism involved vitamin K2 regulating the FAK‐AKT–mTOR‐P70S6K pathway through Ccn2 to promote protein synthesis in skeletal muscle. In T2DM patients, the fortification of vitamin K2 in yogurt for supplementation improved not only the skeletal muscle mass and grip strength but also insulin resistance and blood glucose levels. We thank BioRender (https://BioRender.com) for providing platform support for drawing our research flowchart.
**Table S1.** Primer sequences used for RT‐qPCR.
**Table S2.** The effects of VK2 supplementation on skeletal muscle and biochemical indicators.
**Table S3.** The changes of skeletal muscle and biochemical indicators from baseline to endpoint.
