# Supplementary material for: Vitamin K2 Alleviates Insulin Resistance Associated Skeletal Muscle Atrophy via the AKT/mTOR Signalling Pathway
Source: J Cachexia Sarcopenia Muscle. 2025 Jun 4;16(3):e13840. doi: 10.1002/jcsm.13840 (PMC12134785; doi:10.1002/jcsm.13840)
Supplement: Supplementary file 1 — Figure S1. Vitamin K2 improved the whole‐body energy metabolism in high‐fat diet mice. (A) energy expenditure (kcal/h). (B) Respiratory exchange ratio, n = 3. *p < 0.05, **p < 0.01. Figure S2. Vitamin K2 reduced lipid accumulation induced by high‐fat diet. (A) The relative mRNA expression of nuclear respiratory factor 1 (Nrf1), n = 4. (B) The relative mRNA expression of mitochondrial transcription factor A (Tfam), n = 4. (C) HE staining of liver and epididymal white adipose tissue (eWAT). (D) The weight of epididymal fat, inguinal fat, perirenal fat, and brown fat tissues (g), n = 10. *p < 0.05, **p < 0.01. Figure S3. VK2 alleviated disorders in glucose and lipid metabolism. (A) Fasting glucose at 8 weeks of intervention (mmol/L), n = 10. (B) Serum insulin levels (ng/mL), n = 10. (C) Serum alanine aminotransferase (ALT) levels (U/L), n = 10. (D) Serum aspartate aminotransferase (AST) levels (U/L), n = 10. *p < 0.05, **p < 0.01. Figure S4. (A) The number of differentially expressed genes in the overlap between gene sets of DEGs in the LVK and HVK group in skeletal muscle. (B) GO analysis of DEGs in the HVK groups in skeletal muscle. Figure S5 The analysis and screening of target genes modulated by vitamin K2. (A) Definitions of rescue genes. (B) Target genes identified through PPI and Cytoscape analysis. Four different topological analysis methods, degree, MCC, MNC and EPC were used to extract hub genes by cytoHubba module in Cytoscape. (D) The relative mRNA expression of potential hub genes, n = 4. *p < 0.05, **p < 0.01. Figure S6. Study profile of the randomized controlled trial in T2DM subjects. Figure S7. Vitamin K2 improved skeletal muscle mass and exercise capacity in high‐fat diet induced IR mice while reducing IR and glucolipid disorders. The probable mechanism involved vitamin K2 regulating the FAK‐AKT–mTOR‐P70S6K pathway through Ccn2 to promote protein synthesis in skeletal muscle. In T2DM patients, the fortification of vitamin K2 in yogurt for supplementat [file JCSM-16-e13840-s001.docx]

**Supplementary information for:**

**Vitamin K2 alleviates insulin resistance associated skeletal muscle atrophy via the AKT/mTOR signaling pathway**

**Supplementary Methods**

**Animal dose calculation**

In this study, the choice of doses was based on our previous randomized controlled trial ^[1]^. In that trial, we found that vitamin K2 supplementation in dose of 90 µg/d performed a significant effect on reducing bone loss. This is a relatively low and effective dose compared with the doses used in other studies ^[2-4]^.

Based on the dose of 90 µg/d in human, we calculated the doses administrated to mice. According to previous studies ^[5]^, the dose calculation formula is as follows:

**AED**^a^ **(mg/kg) = Human does (mg/kg) × (Human K_m_**^b^**/Animal K_m_**^c^**)**

^a^ Animal equivalent dose (HED); ^b^ correction factor (K_m_) for human; ^c^ correction factor (K_m_) for mice.

The correction factor (K_m_) is estimated by dividing the average body weight (kg) of species to its body surface area (m^2^). The average human body weight is 60 kg, and the body surface area is 1.62 m^2^. Therefore, the K_m_ factor for human is calculated by dividing 60 by 1.62, which is 37. The average mice weight is 0.02 kg, and the body surface area is 0.007 m^2^. The K_m_ factor for mice is 3.

AED = 90 µg/60 kg × (37/3) = 18.5 µg/kg

To minimize the damage to mice by gavage, we chose to gavage on alternate days. In addition, the half-life of vitamin K2 is approximately 72 hours, and the dose was appropriately increased to ensure an effective dose. Considering the above factors, the final dose formula is as follows:

(18.5^a^ × 2^b^) × (1+1/2)^c^ ≈ 50 µg/kg bw

^a^ The AED dose per day; ^b^ Frequency of gavage (once every two days); ^c^ The dose increased by

taking into account the loss during the half-life; bw, body weigh.

To simultaneously verify the safety of vitamin K2, we increased the dose by 40 times based on the dose of 50 µg/kg bw, which was 2 mg/kg bw. Therefore, in the animal experiments, the two doses of 50 µg/kg bw and 2 mg/kg bw were used.

**Body fat rate measurement of mice**

Body fat rate was measured by a non-invasive Bioimpedance Analyzer (ImpediMed Vet BIS1, Australia). Mice were fasted overnight before measurement. The final result was the average of three consecutive assays.

**RNA-seq**

Sequencing libraries were generated using an NEBNext® UltraTM RNA Library Prep Kit (Illumina), and samples were sequenced on an Illumina Hiseq platform and 150 bp paired-end reads were generated. DESeq2 R package (1.36.1) was used to analyse the differentially expression gene of two groups. The screening criteria were p < 0.05 and the expression change greater than 1.5fold. Gene Ontology (GO) enrichment analysis of differentially expressed genes was implemented by the clusterProfiler R package (3.16). The GO terms with corrected P value < 0.05 were considered significantly enriched by differential expressed genes. PPI analysis of differentially expressed genes was based on the STRING database (12.0), and Cytoscape was used to calculate the scores of the hub genes.

**Western blotting analysis**

Total protein in skeletal muscles and cells was extracted with RIPA buffer (Beyotime) containing phosphatase inhibitors and protease inhibitors. Protein concentration was measured (BCA assay) and normalized with PBS and SDS-PAGE sample loading buffer before being heated for 10 min at 100 ◦C. Protein samples were separated on 6 – 10% Bis-Tris protein gels (Epizyme Biotech, China) and transferred onto polyvinylidene fluoride membranes (PVDF, Millipore, Germany). Membranes were blocked in 3% BSA for 1 h at room temperature and incubated overnight with primary antibodies in Western Antibody Dilution Buffer (Beyotime) at 4 ◦C. Subsequently, membranes were washed 3×10 min in TBS before incubation in secondary antibodies for 1h at 30 ◦C. All blots were imaged using the FluorChem system (Bio-techne).

Primary antibodies: these primary antibodies were obtained from Cell Signaling Technology (CST): Ccn2 (#86641), FAK (#3285), p^Tyr397^FAK (#3283), AKT (#9272), p^S473^AKT (#4058), mTOR (#2972), p^s2448^mTOR (#2971), P70S6K (#34475), p^Thr389^P70S6K (#9234), β-actin (#4970) at a dilution of 1:1000; anti-Puromycin antibody (Sigma-Aldrich, ZMS1016, 1:10000). Secondary antibodies: Anti-Rabbit IgG (Fc), AP Conjugate (Promega, S373B, 1:7500), Anti-rabbit IgG, HRP-linked Antibody (CST, 7074, 1:1000), Anti-mouse IgG, HRP-linked Antibody (CST, 7076, 1:10000).

**Immunostaining**

Skeletal muscle samples were embedded in optimal cutting temperature compound (SAKURA, Tissue-Tek) and snap frozen by liquid nitrogen. Muscle sections (10 µm) were blocked and permeabilized in PBS containing 10% goat serum and 0.4% triton X-100 for 30 min, then incubated with type IIB fiber primary antibody (1:300, DSHB, BF-F3) at 4℃ for 12 h. Sections were washed 3×10 min in PBS, and then incubated with secondary antibody Alexa488 (1:1000, ThermoFisher, A21042) for 2 h at room temperature. Sections were then washed 3×10 min in PBS and mounted with Antifade Mounting Medium (Beyotime, P0126).

For C2C12 myotubes staining, cells were fixed with 4% parafamaldehyde for 15min, and blocked and permeabilized for 30min. Cell samples were incubated with anti-MYHC atibody (Abcam, ab37484, 1:400) at 4℃ overnight and washed 3×10 min in PBS, and then incubated with secondary antibody Alexa647 (Abcam, ab150115, 1:1000).

**Molecular docking**

The structure of VK2 was obtained from the PubChem database (https://pubchem.ncbi.nlm.nih.gov/). The 3D structure of the Ccn2 protein was downloaded from the RCSB Protein Data Bank (https://www.rcsb.org/). Molecules and ligands were were removed from Ccn2 protein by Pymol software, and then the AutoDock Tools 1.5.6 was used for molecular docking. The results were visualized through Discovery Studio and Pymol software.

**Cell viability**

To determine the optimal intervention concentration of PA and VK2, the Cell Counting Kit-8 (CCK-8, MCE) was used to measure the cell viability of differentiated C2C12 myotubes. Myotubes were cultured in a series of PA concentrations (0, 0.1, 0.2, 0.3, 0.4, 0.6, 0.8, 1 and 2 mM) and VK2 concentrations (0, 2.5, 5, 10, 12.5, 25, 50, 75, 100 and 125 µM) for 24 h, respectively. Next, the myotubes were incubated in medium containing CCK8 reagent at 37 °C for 45 min, and the supernatant was collected to detect the absorbance value at 450 nm. The cell viability was calculated as the absorbance ratio of different concentrations of intervention to control.

**Randomized control trial**

**Study design and intervention**

All participants were asked to take one cup of yogurt every evening after dinner and kept the records. The shelf life of yogurt was 21 days. To maintain freshness, yogurt was distributed every two weeks and stored at 4℃. Compliance was estimated by intake records and telephone follow-up. The study was performed in Harbin Medical University, Harbin, China.

**Measurements**

A food-frequency questionnaire was applied to assess the dietary intake of subjects and estimated by using the Food Nutrition Calculator (V1.6; Chinese CDC).

**Sample size**

Considering the realistic benefits of skeletal muscle improvement in patients with type 2 diabetes, the sample size calculation was based on HbA1c change according to a difference (0.5%) which has been widely used in trials testing glucose-lowering medications in patients with type 2 diabetes ^[6]^. Considering a 15% dropout rate, 28 participants were needed in each group (2-sided, 80% power, and α 0.05).

**Supplementary Figures and Tables**


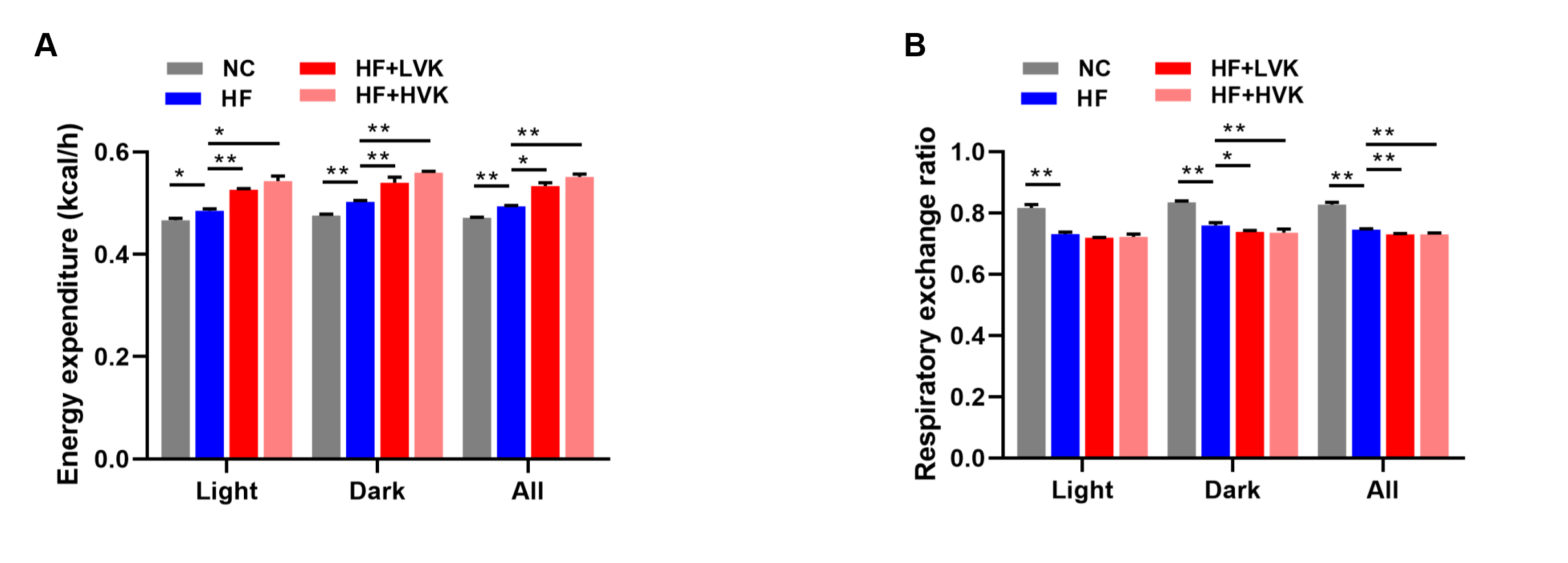


**Figure S1** Vitamin K2 improved the Whole-body energy metabolism in high-fat diet mice. (A) energy expenditure (kcal/h). (B) respiratory exchange ratio, n = 3. **p* <0.05, ***p* <0.01.


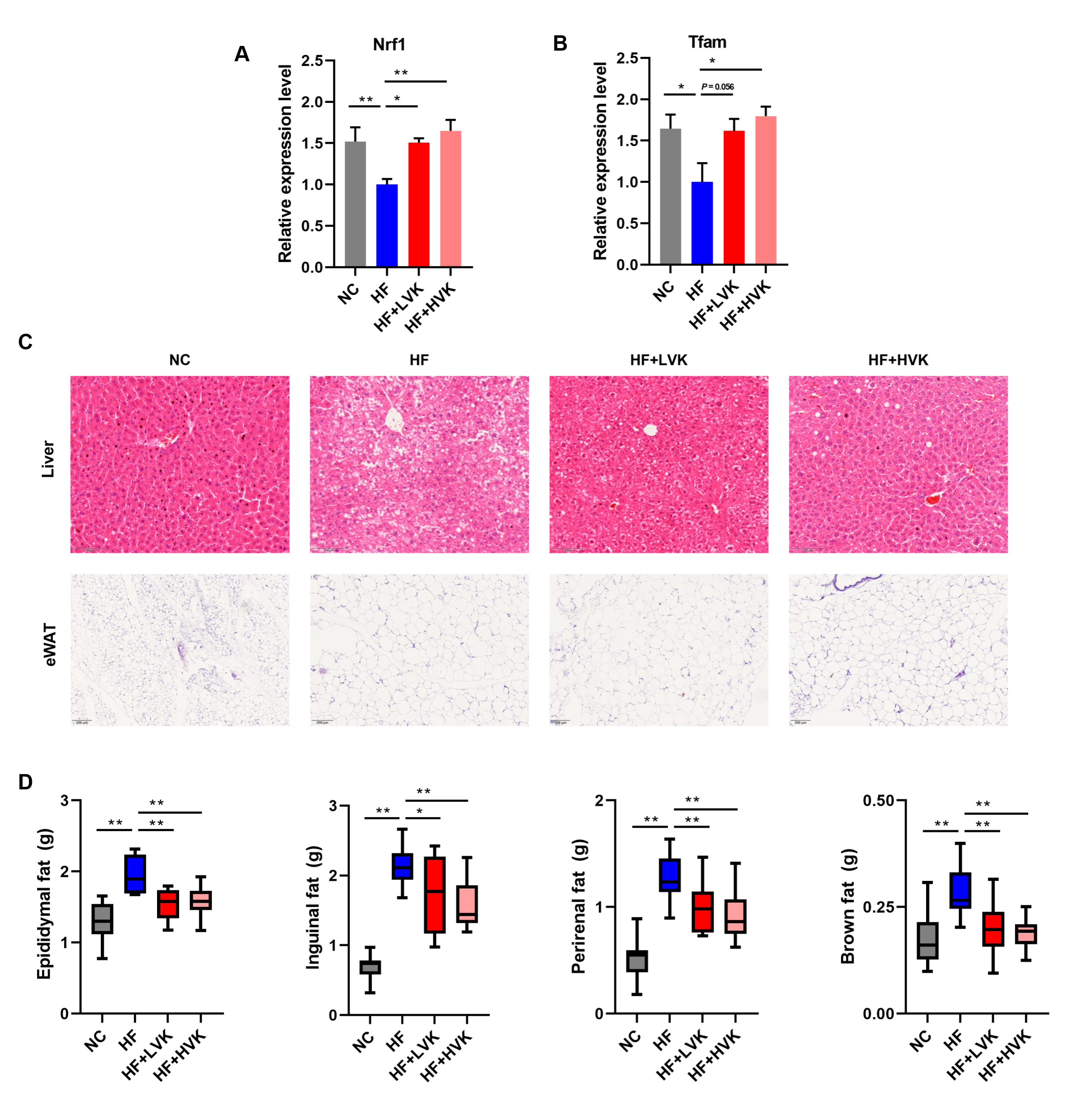


**Figure S2** Vitamin K2 reduced lipid accumulation induced by high-fat diet. (A) The relative mRNA expression of nuclear respiratory factor 1 (Nrf1), n = 4. (B) The relative mRNA expression of mitochondrial transcription factor A (Tfam), n = 4. (C) HE staining of liver and epididymal white adipose tissue (eWAT). (D) The weight of epididymal fat, inguinal fat, perirenal fat, and brown fat tissues (g), n = 10. **p* <0.05, ***p* <0.01.

**
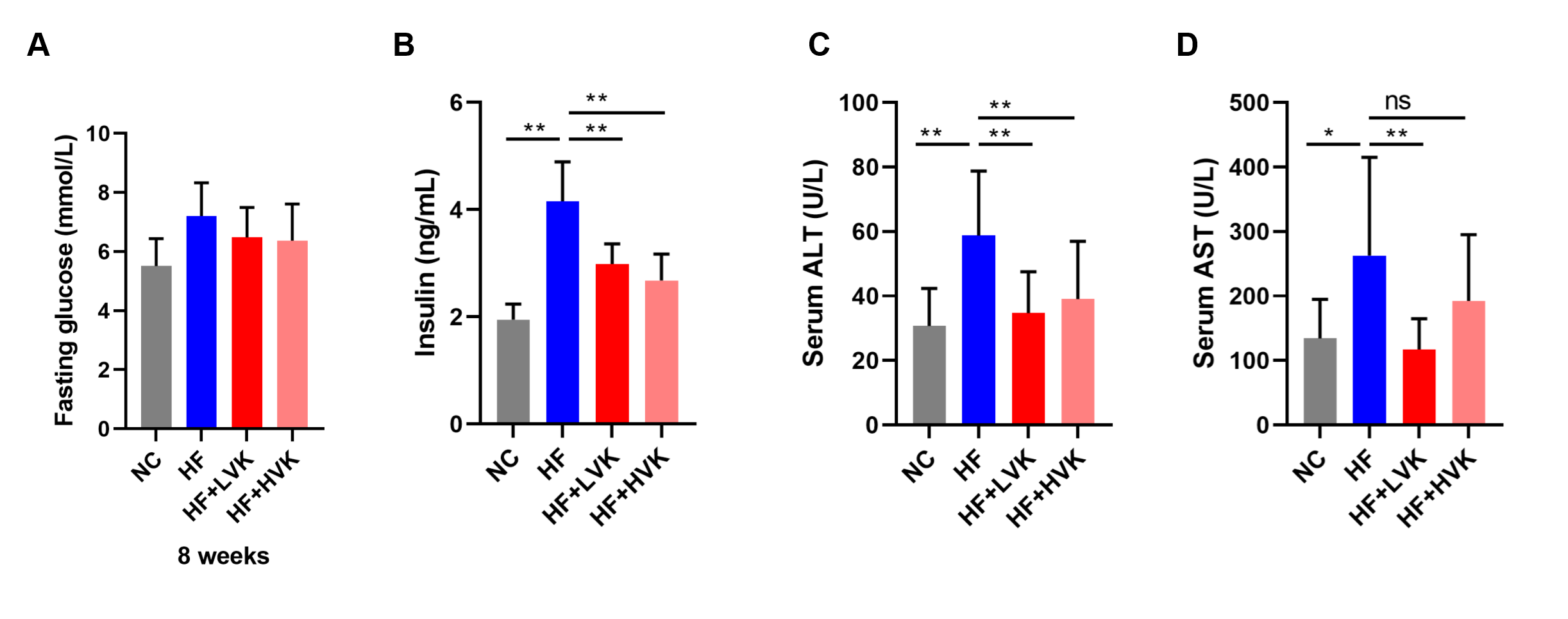
**

**Figure S3** VK2 alleviated disorders in glucose and lipid metabolism. (A) Fasting glucose at 8 weeks of intervention (mmol/L), n = 10. (B) Serum insulin levels (ng/mL), n = 10. (C) Serum alanine aminotransferase (ALT) levels (U/L), n = 10. (D) Serum aspartate aminotransferase (AST) levels (U/L), n = 10. **p* <0.05, ***p* <0.01.

**
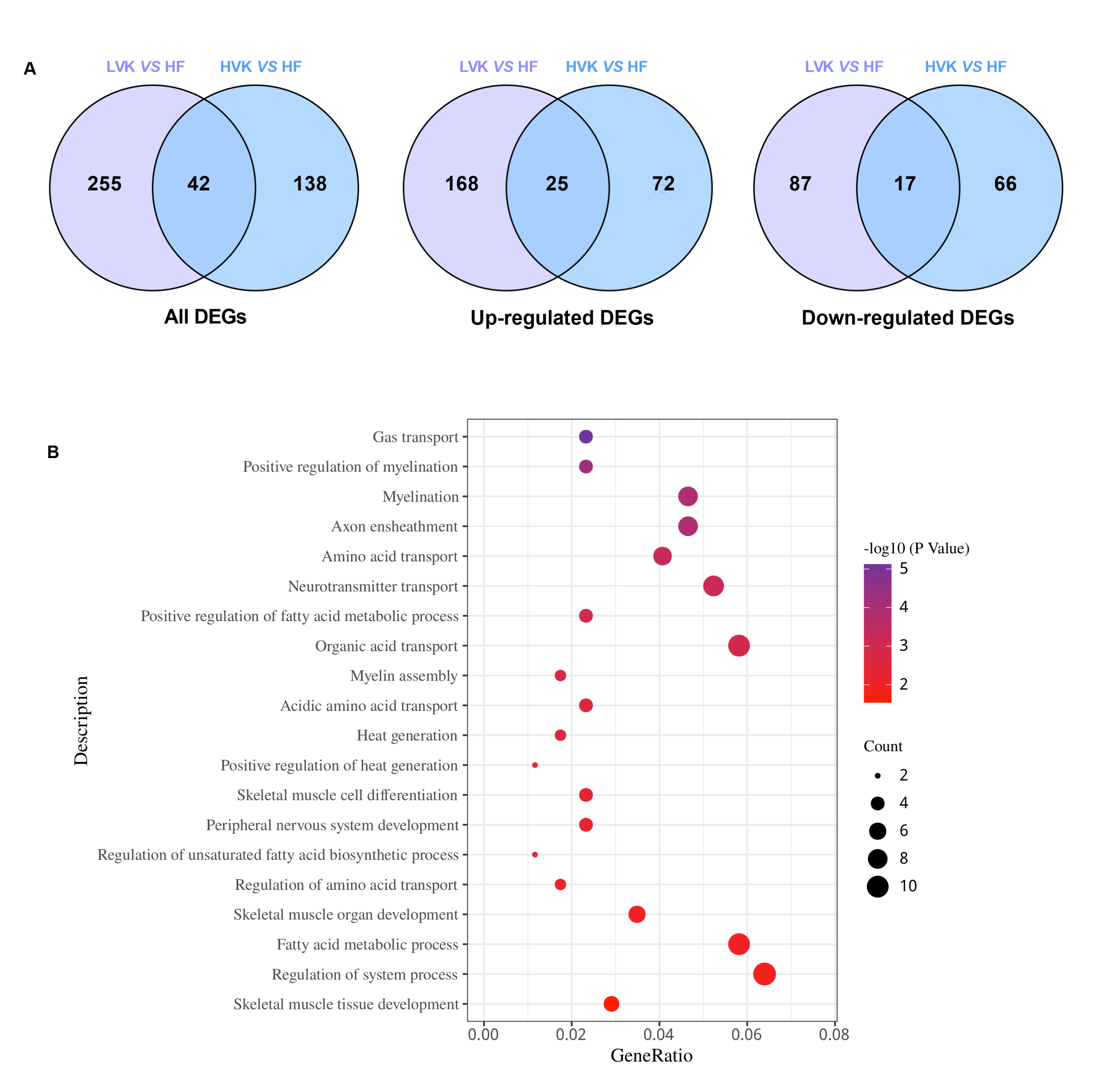
**

**Figure S4** (A) The number of differentially expressed genes in the overlap between gene sets of DEGs in the LVK and HVK group in skeletal muscle. (B) GO analysis of DEGs in the HVK groups in skeletal muscle.

**
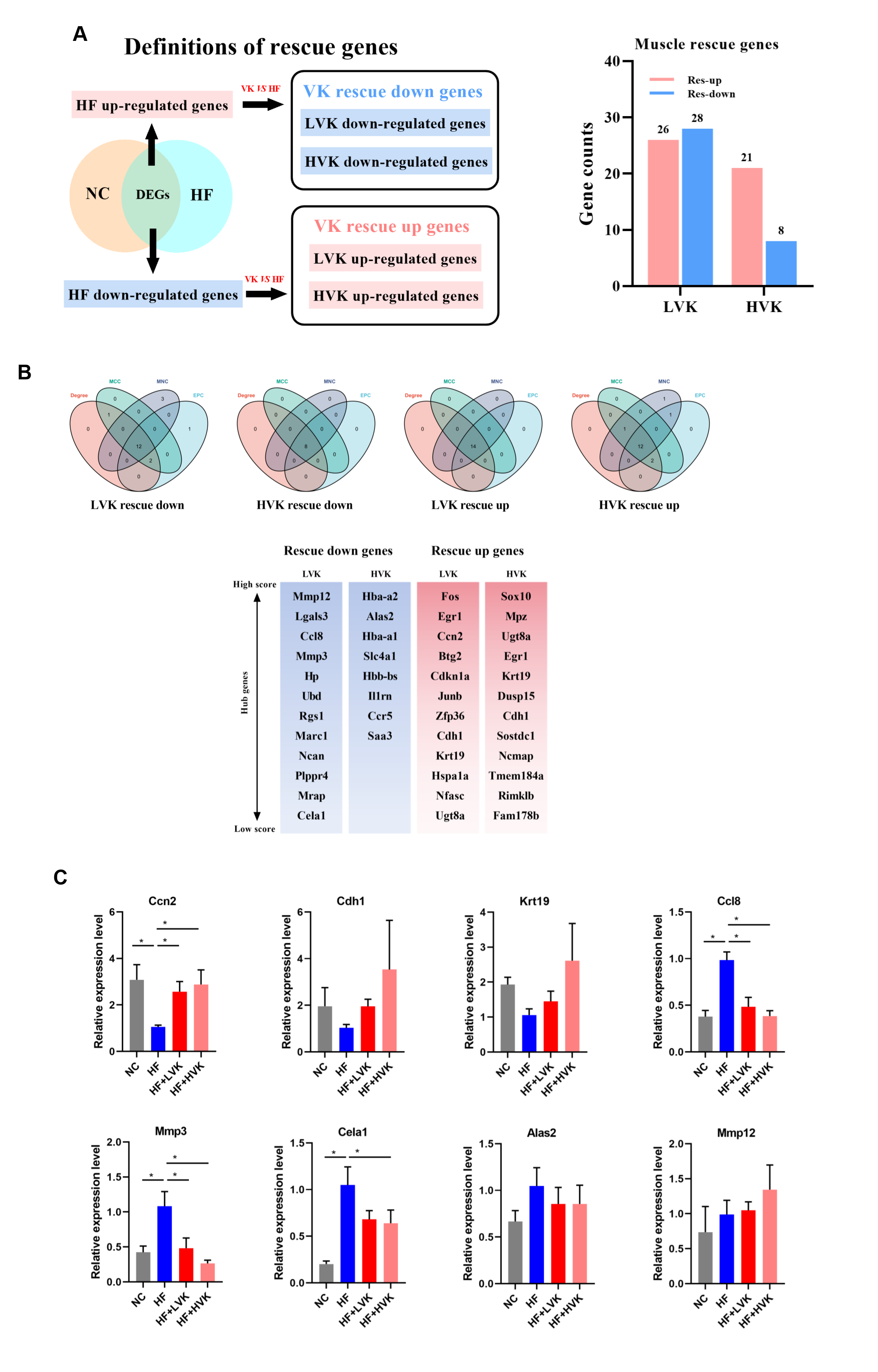
**

**Figure S5** The analysis and screening of target genes modulated by vitamin K2. (A) Definitions of rescue genes. (B) Target genes identified through PPI and Cytoscape analysis. Four different topological analysis methods, degree, MCC, MNC and EPC were used to extract hub genes by cytoHubba module in Cytoscape. (D) The relative mRNA expression of potential hub genes, n = 4. **p* <0.05, ***p* <0.01.

**
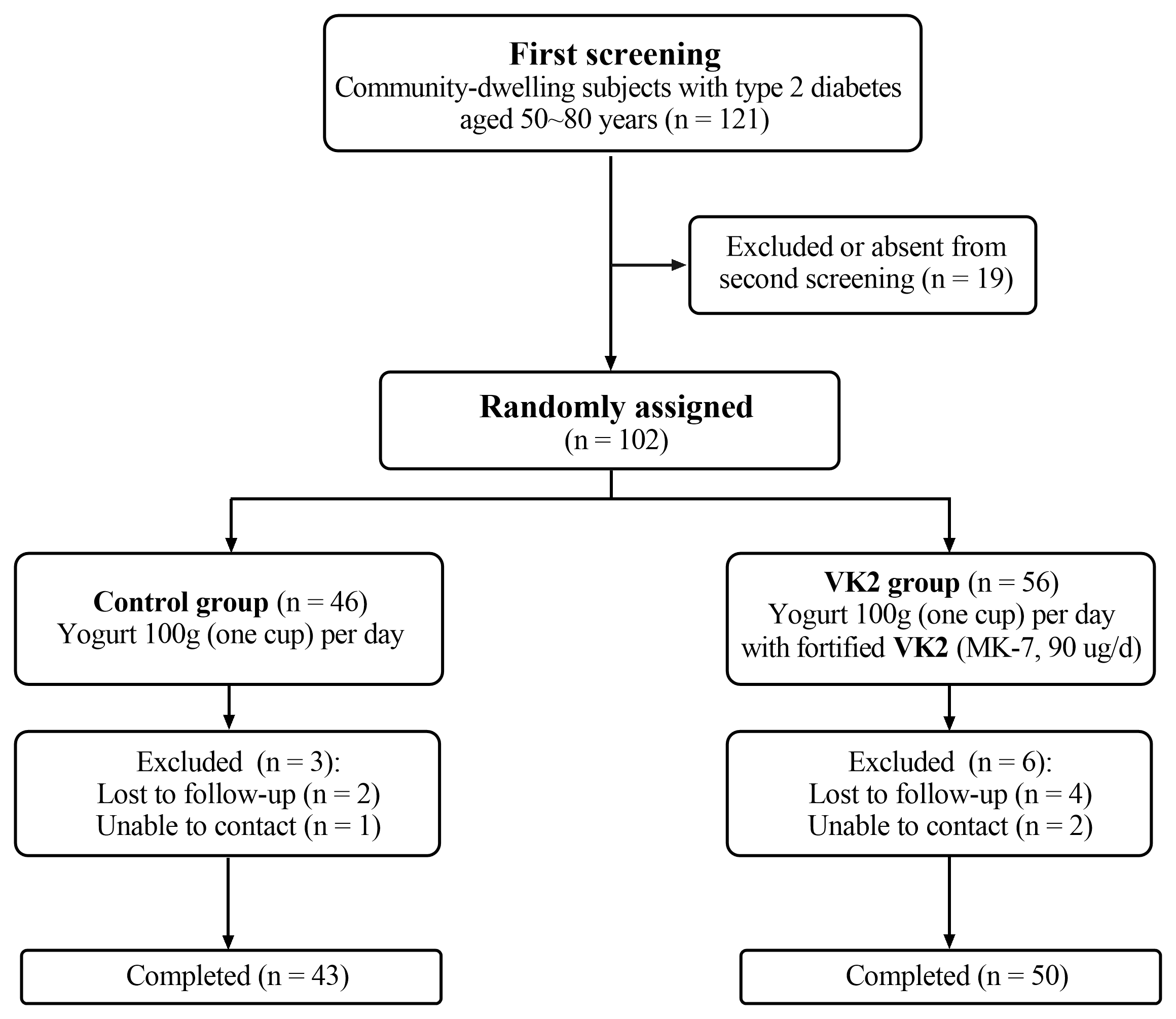
**

**Figure S6** Study profile of the randomized controlled trial in T2DM subjects

**
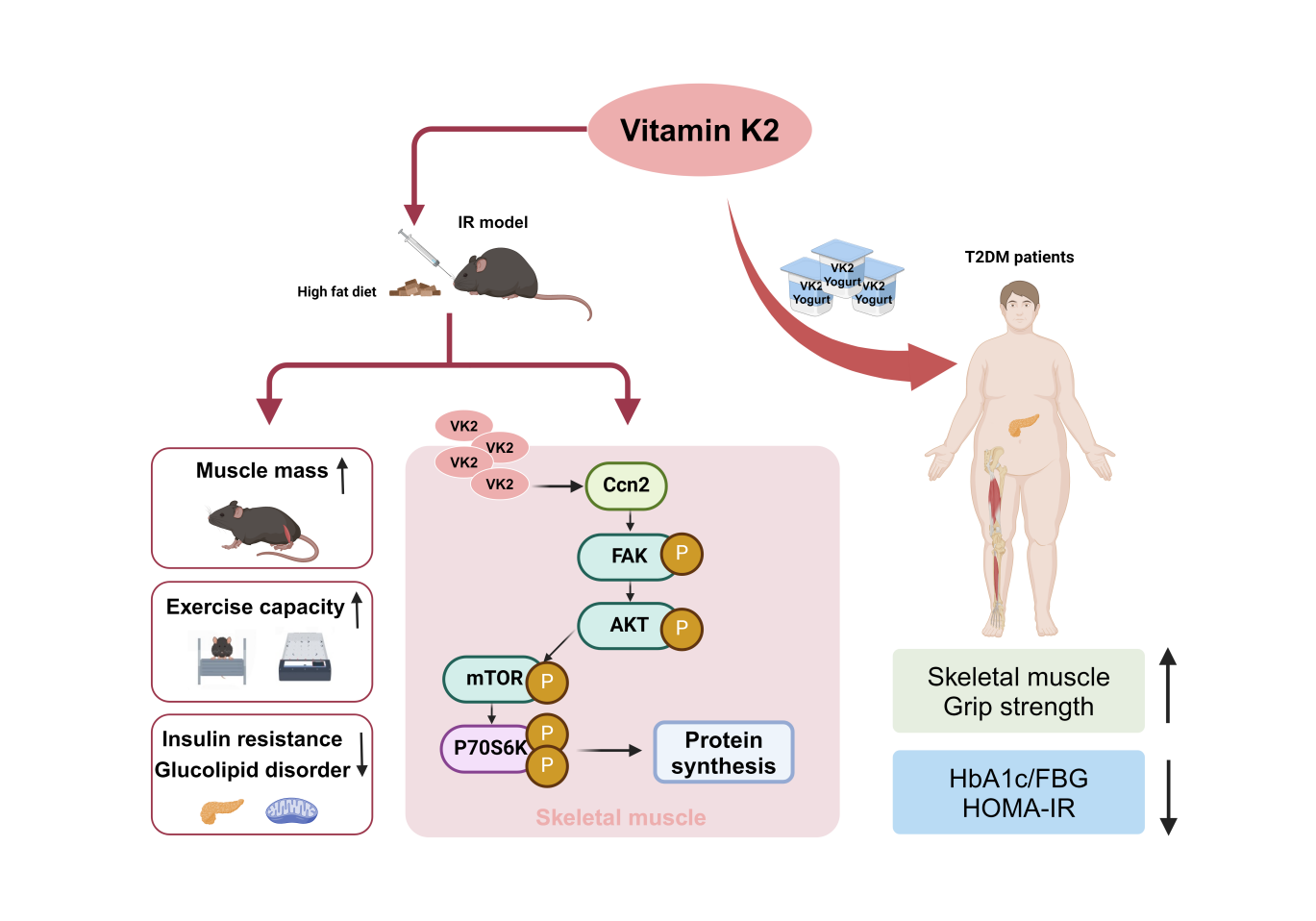
**

**Figure S7** Vitamin K2 improved skeletal muscle mass and exercise capacity in high-fat diet induced IR mice while reducing IR and glucolipid disorders. The probable mechanism involved vitamin K2 regulating the FAK-AKT-mTOR-P70S6K pathway through Ccn2 to promote protein synthesis in skeletal muscle. In T2DM patients, the fortification of vitamin K2 in yogurt for supplementation improved not only the skeletal muscle mass and grip strength but also insulin resistance and blood glucose levels. We thank BioRender (https://BioRender.com) for providing platform support for drawing our research flowchart.

**Table S1** Primer Sequences used for RT-qPCR.

|  | **Forward primer sequences** | **Reverse primer sequences** |
| --- | --- | --- |
| Ccn2 | AGAACTGTGTACGGAGCGTG | GTGCACCATCTTTGGCAGTG |
| Cdh1 | GCTGGACCGAGAGAGTTACC | ACGTGCTTGGGTTGAAGACA |
| Krt19 | AGGTCAGTGTGGAGGTGGATTC | GCAAGGCGTGTTCTGTCTCA |
| Mmp12 | TCTGCTGAAAGGAGTCTGCAC | AGGTTTCTGCTGGGAACCTTCAG |
| Ccl8 | AAGCTGAAGATCCCCCTTCG | CTGCTTGGTCTGGAAAACCACA |
| Mmp3 | GGCGCAAATCTCTCAGGACT | AGCCCAGAACTGATTTCCTTT |
| Cela1 | TCTTGGCCGTCTCAGATTTCC | CACCCAGTTGCTTCGGATGA |
| Alas2 | AGGCTGGAGGAGAACTCCAA | TGCTCAGCAGGTCTGTCTTG |
| Nrf1 | TCTGCTGTGGCTGATGGAGAGG | GATGCTTGCGTCGTCTGGATGG |
| Tfam | CGTATTGCGTGAGACGAACC | CTTCGGAATACAGACAAGACTGA |
| β-actin | AAGGCCAACCGTGAAAAGAT | GTGGTACGACCAGAGGCATAC |

**Table S2** The effects of VK2 supplementation on skeletal muscle and biochemical indicators.

|  |  | **Placebo group** | **VK2 group** | ***P*_treatment×time_** |
| --- | --- | --- | --- | --- |
| **Grip strength (Kg)** | month 0 | 30.07 ± 9.71 | 29.24 ± 8.75 |  |
|  | month 3 | 29.23 ± 9.12 | 29.66 ± 8.86 |  |
|  | month 6 | 28.15 ± 8.57 | 31.17 ± 9.35 | 0.017 |
|  |  |  |  |  |
| **6m-GS (m/s)** | month 0 | 1.14 ± 0.16 | 1.09 ± 0.15 |  |
|  | month 3 | 1.14 ± 0.15 | 1.09 ± 0.15 |  |
|  | month 6 | 1.12 ± 0.16 | 1.1 ± 0.15 | 0.573 |
|  |  |  |  |  |
| **ASM (Kg)** | month 0 | 29.78 ± 6.33 | 28.82 ± 5.61 |  |
|  | month 3 | 29.5 ± 6.32 | 29.64 ± 5.22 |  |
|  | month 6 | 29.24 ± 6.35 | 30.46 ± 5.44 | 0.001 |
|  |  |  |  |  |
| **ASMI (Kg/m2)** | month 0 | 10.95 ± 1.39 | 10.62 ± 1.33 |  |
|  | month 3 | 10.84 ± 1.34 | 10.83 ± 1.09 |  |
|  | month 6 | 10.84 ± 1.32 | 11.19 ± 1.25 | <0.001 |
|  |  |  |  |  |
| **HbA1c (%)** | month 0 | 7.83 ± 1.25 | 7.86 ± 1.23 |  |
|  | month 3 | 7.94 ± 1.27 | 7.34 ± 1.15 |  |
|  | month 6 | 8.02 ± 1.1 | 7.02 ± 1.13 | <0.001 |
|  |  |  |  |  |
| **FBG (mmol/L)** | month 0 | 8.22 ± 2.68 | 8.06 ± 2.67 |  |
|  | month 3 | 8.27 ± 2.7 | 7.83 ± 2.6 |  |
|  | month 6 | 8.48 ± 2.73 | 7.52 ± 2.53 | 0.056 |
|  |  |  |  |  |
| **FINS (μU/mL)** | month 0 | 10.99 ± 2.77 | 11.26 ± 2.22 |  |
|  | month 3 | 10.69 ± 2.69 | 9.57 ± 1.89 |  |
|  | month 6 | 9.79 ± 4.03 | 7.79 ± 1.81 | <0.001 |
|  |  |  |  |  |
| **HOMA-IR** | month 0 | 4.00 ± 1.59 | 4.02 ± 1.48 |  |
|  | month 3 | 3.91 ± 1.56 | 3.32 ± 1.23 |  |
|  | month 6 | 3.75 ± 2.17 | 2.62 ± 1.1 | <0.001 |

The data are expressed as means ± SEs.

Abbreviations: 6m-GS, six-meter gait speed; SM, skeletal muscle; SMI, skeletal muscle index; FBG, fasting blood glucose; FINS, fasting insulin; HOMA-IR, homeostasis model of assessment insulin resistance.

*P*_treatment×time_ values were based on linear mixed model (placebo group, n = 43, VK2 group, n = 50).

**Table S3** The changes of skeletal muscle and biochemical indicators from baseline to endpoint.

|  | **Placebo group** | | **VK2 group** | |  |
| --- | --- | --- | --- | --- | --- |
|  | **Means ± SDs** | **Medians (IQRs)** | **Means ± SDs** | **Medians (IQRs)** | ***P*** |
| **Grip strength (Kg)** | -1.92 ± 4.15 | -2.3 (-3.76, 0.56) | 1.93 ± 2.28 | 2.28 (1.11, 3.24) | <0.001 |
| **6m-GS (m/s)** | -0.02 ± 0.11 | -0.03 (-0.08, 0.02) | 0.01 ± 0.06 | 0.01 (-0.01, 0.05) | 0.007 |
| **ASM (Kg)** | -0.53 ± 1.88 | -0.26 (-0.86, 0.52) | 1.64 ± 1.77 | 1.45 (0.28, 2.49) | <0.001 |
| **ASMI (Kg/m2)** | -0.1 ± 0.41 | -0.08 (-0.3, 0.19) | 0.57 ± 0.7 | 0.49 (0.04, 0.89) | <0.001 |
| **HbA1c (%)** | 0.18 ± 1.27 | 0.3 (-0.7, 0.9) | -0.83 ± 1.41 | -0.33 (-2.01, 0.2) | <0.001 |
| **FINS (μU/mL)** | -1.2 ± 3.65 | -1.72 (-3.98, 0.77) | -3.47 ± 2.27 | -3.27 (-4.83, -2.06) | 0.004 |
| **FBG (mmol/L)** | 0.25 ± 2.08 | 0.26 (-0.61, 1.17) | -0.54 ± 1.9 | -0.49 (-1.37, 0.31) | 0.058 |
| **HOMA-IR** | -0.21 ± 1.93 | -0.49 (-1.71, 0.66) | -1.39 ± 1.01 | -1.29 (-1.92, -0.65) | 0.002 |

Abbreviations: 6m-GS, six-meter gait speed; SM, skeletal muscle; SMI, skeletal muscle index; FBG, fasting blood glucose; FINS, fasting insulin; HOMA-IR, homeostasis model of assessment insulin resistance.

*P* values were assessed by ANOVA (changes of SM, HbA1c and FBG) and Wilcoxon test (changes of grip strength, 6m-GS, SMI, FINS and HOMA-IR) (placebo group, n = 43, VK2 group, n = 50).

**Abbreviations**

IR Insulin resistance

VK2 Vitamin K2

HFD High-fat diet

T2DM Type 2 diabetes mellitus

OGTT Oral glucose tolerance test

RCT Randomized controlled trial

SM Skeletal muscle mass

SMI Skeletal muscle mass index

6-m GS 6-meter gait speed

HbA1c Hemoglobin A1c

Dp-ucMGP Dephosphorylated uncarboxylated matrix Gla protein

HOMA-IR Homeostasis model of assessment insulin resistance

Ccn2 Cellular communication network factor 2

FAK Focal adhesion kinase

P70S6K Ribosomal protein S6 kinase

**Reference**

1. Zhang, Y., Z. Liu, L. Duan, Y. Ji, S. Yang, Y. Zhang, H. Li, Y. Wang, P. Wang, J. Chen and Y. Li, Effect of Low-Dose Vitamin K2 Supplementation on Bone Mineral Density in Middle-Aged and Elderly Chinese: A Randomized Controlled Study. Calcif Tissue Int, 2020, 106(5):476-485.

2. Knapen, M.H., N.E. Drummen, E. Smit, C. Vermeer and E. Theuwissen, Three-year low-dose menaquinone-7 supplementation helps decrease bone loss in healthy postmenopausal women. Osteoporos Int, 2013, 24(9):2499-507.

3. Ronn, S.H., T. Harslof, S.B. Pedersen and B.L. Langdahl, Vitamin K2 (menaquinone-7) prevents age-related deterioration of trabecular bone microarchitecture at the tibia in postmenopausal women. Eur J Endocrinol, 2016, 175(6):541-549.

4. Emaus, N., C.G. Gjesdal, B. Almas, M. Christensen, A.S. Grimsgaard, G.K. Berntsen, L. Salomonsen and V. Fonnebo, Vitamin K2 supplementation does not influence bone loss in early menopausal women: a randomised double-blind placebo-controlled trial. Osteoporos Int, 2010, 21(10):1731-40.

5. Nair, A.B. and S. Jacob, A simple practice guide for dose conversion between animals and human. J Basic Clin Pharm, 2016, 7(2):27-31.

6. Little, R.R., C.L. Rohlfing, D.B. Sacks and C. National Glycohemoglobin Standardization Program Steering, Status of hemoglobin A1c measurement and goals for improvement: from chaos to order for improving diabetes care. Clin Chem, 2011, 57(2):205-14.
